# Miau, a microbalance autosampler

**DOI:** 10.1016/j.ohx.2021.e00215

**Published:** 2021-06-25

**Authors:** Matheus C. Carvalho

**Affiliations:** Centre for Coastal Biogeochemistry Research, Environmental Analysis Laboratory, Southern Cross Analytical Research Services, Southern Cross University, PO Box 157, Lismore 2480, NSW, Australia

**Keywords:** 3D printing, Arduino, AutoIt, Closed loop, elemental analysis, Laboratory automation, OpenSCAD, Pick and place, Sampling, Stable isotopes

## Abstract

Powder weighing is an essential but tedious activity in many branches of science. Here I describe a MIcrobalance AUtosampler (miau) that transfers solids in the sub-mg range to a microbalance. Miau is a pick-and-place machine which moves a gripper with dual function: 1) move tin capsules; 2) deliver powder from a container to tin capsules. In our laboratory we routinely use miau to prepare working standards for quality control of elemental and isotopic analyses. In a test, miau produced standards between 0.3 and 1.1 mg, which is a useful range in our laboratory. Failure to produce a weighed standard happened in 5% of the cases. A comparison with manual measurements demonstrated that obtained amounts for automated samples were as accurate and precise as manually prepared ones. Setup for daily use is simple, and the microbalance can be easily used alternately with or without miau. Miau is a low-cost device that can work with microbalances from many manufacturers, and can be readily adopted by many laboratories.

## Graphical abstract

**Figure:**
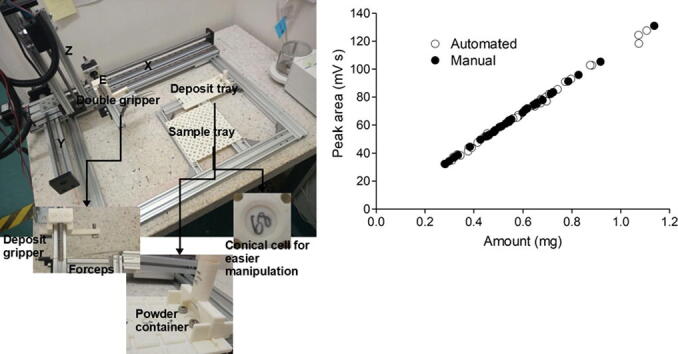

## Introduction

### Specifications table

**Table:** 

Hardware name	Miau
Subject area	• Chemistry and Biochemistry • Environmental, Planetary and Agricultural Sciences • Medicine, Forensics
Hardware type	• Solid sample handling
Open Source License	GNU General Public License (GPL) 3.0
Cost of Hardware	AU$ 900 (does not include microbalance and computer)
Source File Repository	https://osf.io/y2hu8/ or https://doi.org/10.17605/OSF.IO/Y2HU8

## Hardware in context

Weighing is one of the most fundamental scientific measurements, being performed in most scientific laboratories. In many cases, the substance being weighed is a powder. Manual powder weighing is work-intensive and time consuming. A person weighing powders cannot perform any other activity while weighing. Therefore, if there is a need for the repeated weighing of a large number of samples, it is likely that dedicated staff will be allocated to this task in some places. From the viewpoint of the supply chain for chemicals in the pharmaceutical and biotechnological industries, there are numerous stablished automated solutions for storage and retrieval of powders, and weighing remains the bottleneck [1].

Weighing powders is an activity which is simple for a person, but hard for a robot. As such, it is not a strong candidate for automation, because the human workforce dealing with the task does not need to be highly specialized, whereas the robots dealing with it need to be very complex [2]. The main reason for this situation is that weighing powders demands manual dexterity, along visual and tactile perception to deal with different kinds of powders. It could be argued that in some situations even the manual task is hard, for example, when dealing with powders susceptible to statics. However, in most cases dealing with powders for weighing is fairly simple, and a person with minimal training can perform the job. On top of that, available commercial solutions are often very expensive and still not entirely satisfactory [3], and one third of companies in the pharmaceutical and biotechnological industries have acknowledged that manual weighing is indeed a bottleneck in some processes, but they are nonetheless happy to live with the problem [3].

Despite these difficulties, there are numerous commercial devices that perform automated weighing [3]. Existing commercial automated solutions for powder weighing can be grouped in four different types [3]: 1) Controlled flow of solid (mechanically induced, via Archimedes screw, tapping, vibrating or shaking) from a container to a vessel positioned on a balance; 2) Moving balance / powder probe that delivers the powder from containers to vials; 3) Solid pipettes; 4) Statics based powder transfer. Each method has advantages and weaknesses, and sometimes combinations of two or more are employed depending on the application.

One of the main challenges when implementing an automated powder weighing solution is dealing with heterogeneous materials [1], [3]. Thus, the task can be made potentially much simpler if only a single material of known properties is dealt with. In some situations, what is needed consists into weighing the same powder over and over again, for example, when preparing standards for the quality control of elemental and isotopic analyses [4], [5]. Therefore, a machine that could automate this task would be helpful, and potentially not so complex as other devices designed to deal with many different types of powders.

Here I present a MIcrobalance AUtosampler (MIAU) that delivers a chosen powder in a narrow range of masses (around 1 mg) to containers that are individually weighed by a microbalance. This way, a large number of standards can be automatically prepared for elemental and isotopic measurements of solids. Miau can be integrated to any microbalance as long as the balance can be operated using a computer, which makes it readily available to many laboratories. On top of that, miau costs much less than commercial counterparts (less than US$1,000 versus tens to hundreds of thousands of dollars [1]).

## Hardware description

Miau is a cartesian robot, which means that movements are performed on straight lines along three-dimensional axes perpendicular to each other. Due to the widespread availability of their basic components, including control boards, mechanical parts, and 3D printed parts [6], [7], many such cartesian robots have been presented recently in the literature for varied purposes in laboratory [8], [9], [10], [11], [12], [13], [14], [15], [16], [17], [18], [19], [20], [21] and even in the field [22], [23], [24], but none of them specifically for dealing with sample weighing.

**Fig. 1:**
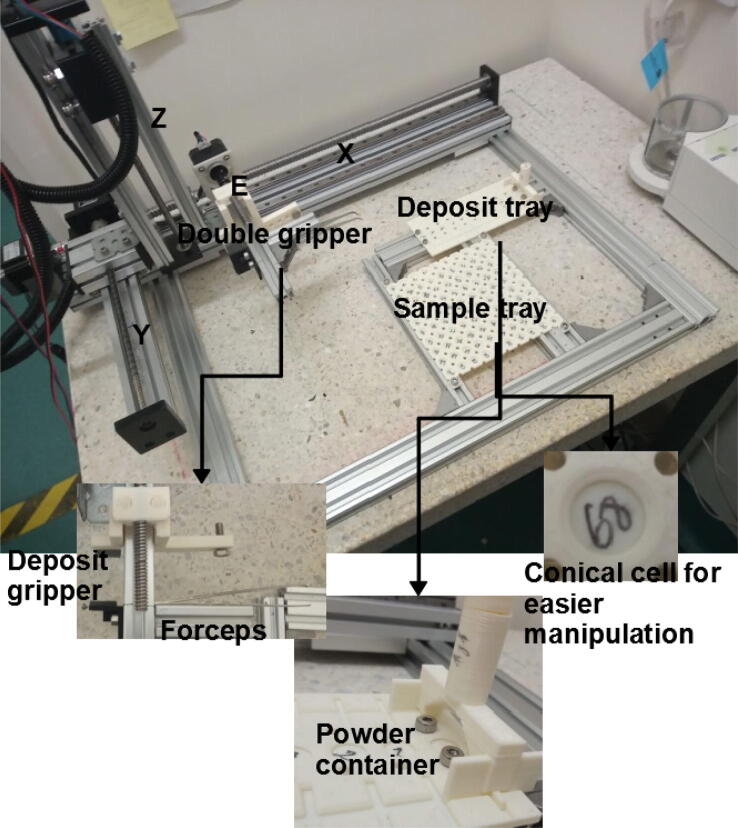
Miau. Lose letters (X, Y Z and E) indicate the movement axes.

More specifically, like a previously presented autosampler [25], miau is a pick and place (pnp) machine. Its gripper has dual function: 1) to actuate forceps that handle tin capsules; 2) to grab a powder container (Fig. 1). Miau also includes a base, where two trays, one for the tin capsules, and another for the sample deposits and powder container, are fixed (Fig. 1). Finally, there is the powder container itself, which is specifically designed to deliver powder in the desired range (Fig. 1).

Linear motion is obtained by the use of linear actuators consisting in leadscrews actuated by stepper motors. The motors are controlled using a computer via a control board operating on the Marlin firmware which enables the use of G-code for motor control [8], [9], [11], [22], [10]. All movements are synchronized to a computer controlled balance (in this case, a microbalance) using the AutoIt scripting language [26]. Although home-made actuators can potentially be built [27], [11], which could reduce the cost of the machine, commercially available actuators were purchased because they cost relatively little, have reliable performance, and are easier to assemble than home-made ones.

Miau performs two tasks: 1) tin capsule manipulation; 2) powder transfer. The first task, tin capsule manipulation, is done without severely deforming the capsule. This is achieved by careful and very precise (error of 0.1 mm) movement. The end effector used for this task are forceps designed for this task being done manually (Fig. 1).

The second task, powder delivery, must achieve powder transfer from the container to the tin capsule inside an acceptable range. The powder container used here has a single hole which, when the container is at rest, does not allow the passage of powder. However, when the container vibrates, powder trickles down the hole. As a general rule, the more the container vibrates, the larger the amount of powder that trickles. The container also has “feet” which allow it to rest stable on its tray (Fig. 1), which by its turn is also designed with grooves to allow easy vertical movement in and out of it. In the weighing procedure, vibration of the powder container is achieved by dropping it from a few mm above the tin capsule to receive the powder. Thus, this powder delivery mechanism can be classified as of “type 1”, that is, mechanically controlled powder flow (see introduction).

The tray for tin capsules was also designed in a way to facilitate manual positioning of the capsules, and reliable automated manipulation using the forceps. It is a very shallow tray, which allows for easy manipulation with the forceps (Fig. 1). Also, the upper part of each cell is in the shape of an inverted truncated cone, which facilitates the return of the tin capsule to its position when delivered by miau (Fig. 1). The tray was designed for 5 × 9 mm tin capsules provided by Sercon (part number SC1276). Different capsules may require a different tray. Only capsules with reasonably flat bottoms can be used, and “shallow” capsules are easier to operate than “deep” ones (due to more favorable center of gravity). If a capsule is placed manually on a flat surface and does not stand by itself, it cannot be used for the procedure. Also, the forceps may fail to catch deformed capsules, and thus it is recommended that capsules as “normal” as possible be used for the procedure.

A video showing miau working is presented in Supplementary Information 1.

## Design files

### Design files summary

Here are included all parts that need to be 3D printed. Some items need heavy infill because they are used in a linear mechanism. The others can be printed with less infill as they do not undergo heavy stress. All files located at https://osf.io/y2hu8/ or https://doi.org/10.17605/OSF.IO/Y2HU8.

**Table:** 

Design file name	File type	Open source license	Recommended infill
Linear carriage and half gripper	STL	GNU General Public License (GPL) 3.0	95%
Stepper motor mount	OpenSCAD	GNU General Public License (GPL) 3.0	95%
Half gripper for powder container	OpenSCAD	GNU General Public License (GPL) 3.0	95%
Support for forceps	OpenSCAD	GNU General Public License (GPL) 3.0	95%
Tray for tin capsules	OpenSCAD	GNU General Public License (GPL) 3.0	20%
Tray for powder container and tin capsules	OpenSCAD	GNU General Public License (GPL) 3.0	20%
Powder container	OpenSCAD	GNU General Public License (GPL) 3.0	20%
Powder container lid	OpenSCAD	GNU General Public License (GPL) 3.0	20%

## Bill of materials

Freight costs are not included in this table. They can be significant for bulky items, like extrusion profiles and linear actuators. Also, the costs are for the machine built here; depending on the application, axes sizes can be made smaller, which can reduce costs.

**Table:** 

Figure or reference	Component	Number	Cost per unit –AU$	Total cost –AU$	Source of materials	Material type
Fig. 2, Fig. 9, Fig. 21	T slot 20x20mm, 250 mm	2	$3.55	$7.10	https://www.aliexpress.com/item/1005001287672245.html	Aluminum
Fig. 13, Fig. 19, Fig. 20	T slot 20x20mm, 200 mm	1	$2.85	$2.85	https://www.aliexpress.com/item/1005001287672245.html	Aluminum
Fig. 12	T slot 20x20mm, 100 mm	1	$1.78	$1.78	https://www.aliexpress.com/item/1005001287672245.html	Aluminum
Fig. 3, Fig. 4, Fig. 5	T slot 20x40mm, 500 mm	3	$10.50	$31.50	https://www.aliexpress.com/item/32813630598.html	Aluminum
Fig. 21	T slot 20x40mm, 250 mm	1	$6.06	$6.06	https://www.aliexpress.com/item/32813630598.html	Aluminum
Fig. 21	T slot 20x40mm, 200 mm	1	$5.66	$5.66	https://www.aliexpress.com/item/32813630598.html	Aluminum
Fig. 5, Fig. 13, Fig. 21	2028 bracket, bag with 20	1	$8.18	$8.18	https://www.aliexpress.com/item/32642711369.html	Aluminum
Fig. 7, Fig. 8, Fig. 10, Fig. 20	Long bracket	2	$1.95	$3.90	https://www.bunnings.com.au/carinya-50-x-50-x-40-x-2mm-make-a-bracket_p3960589	Steel
Fig. 4, Fig. 6, Fig. 8, Fig. 23	Linear actuator, 700 mm, ball screw, with extra rail	1	$450.00	$450.00	https://www.fuyuautomation.com	
Fig. 6, Fig. 7, Fig. 8, Fig. 23	Linear actuator, 200 mm, leadscrew	2	$86.00	$172.00	https://www.ebay.com.au/itm/Lead-Screw-CNC-Linear-Slide-Stage-Stroke-200mm-500mm-42-Actuator-Stepper-Motor/323373368523	
Fig. 14, Fig. 15	NEMA 17 stepper motor with cable	1	$8.65	$8.65	https://www.aliexpress.com/item/4000130492082.html	Metal, plastic
Fig. 14	Shaft coupler, 5 mm to 8 mm	1	$2.21	$2.21	https://www.aliexpress.com/item/4000196552485.html	Metal
Fig. 16	Leadscrew, 100 mm, lead 2 mm, diameter 8 mm	1	$3.50	$3.50	https://www.aliexpress.com/item/32507277503.html	Metal
Fig. 15	M3 screws, 6 mm length, pack with 10	1	$7.20	$7.20	https://www.ebay.com.au/itm/M3-M4-M5-M6-M8-Socket-Head-Cap-Screw-Stainless-Steel-304-Metric-Coarse/142732934590	Metal
Fig. 7, Fig. 10, Fig. 21	M4 screws, 8 mm length, pack with 20	1	$10.20	$10.20	https://www.ebay.com.au/itm/M3-M4-M5-M6-M8-Socket-Head-Cap-Screw-Stainless-Steel-304-Metric-Coarse/142732934590	Metal
Fig. 4	M4 screws, 20 mm length, pack with 10	1	$8.00	$8.00	https://www.ebay.com.au/itm/M3-M4-M5-M6-M8-Socket-Head-Cap-Screw-Stainless-Steel-304-Metric-Coarse/142732934590	
Fig. 7, Fig. 10, Fig. 11, Fig. 13, Fig. 20, Fig. 21	M5 screws, 10 mm length, pack with 50	1	$17.00	$17.00	https://www.ebay.com.au/itm/M3-M4-M5-M6-M8-Socket-Head-Cap-Screw-Stainless-Steel-304-Metric-Coarse/142732934590	Metal
Together with 2028 brackets	Washer for M5 screw, pack with 20	1	$5.80	$5.80	https://www.ebay.com.au/itm/142552908688	Metal
Together with 2028 brackets, and others	Hammer nut for M5 screw, pack with 10	3	$11.00	$33.00	https://www.ebay.com.au/itm/M3-M4-M5-M6-T-Hammer-Nuts-Drop-In-T-Nut-for-3030-Series-Aluminum-Profile/352643731421	Metal
Fig. 10, Fig. 21	Hammer nut for M4 screw, pack with 10	1	$12.13	$12.13	https://www.ebay.com.au/itm/M3-M4-M5-M6-T-Hammer-Nuts-Drop-In-T-Nut-for-3030-Series-Aluminum-Profile/352643731421	Metal
Fig. 9	M5 screws, 30 mm length, pack with 5	1	$5.00	$5.00	https://www.ebay.com.au/itm/M3-M4-M5-M6-M8-Socket-Head-Cap-Screw-Stainless-Steel-304-Metric-Coarse/393027020255	Metal
Fig. 17	M6 screw, 20 mm length, pack with 5	1	$6.80	$6.80	https://www.ebay.com.au/itm/M5-M6-M8-M10-M12-M16-Hex-Head-Set-Screw-Stainless-Steel-304-Metric-Coarse/142560735415	Metal
Fig. 13, Fig. 17	Foam for gripper	1	$7.00	$7.00	https://www.ebay.com.au/itm/5–10-20M-Rectangle-Weather-Stripping-Sponge-Foam-Rubber-Strip-Tape-Door-Seal15mm/293563012819	
Fig. 11	Cable ties, 3 × 100 mm, pack with 100	1	$1.10	$1.10	https://www.aliexpress.com/item/4000235072438.html	
Fig. 11, Fig. 12	Forceps, curved point	1	$2.35	$2.35	https://www.aliexpress.com/item/32908341312.html	Metal
Fig. 22	MKS Gen L control board	1	$17.54	$17.54	https://www.aliexpress.com/item/32969933046.html	Metal, plastic
Fig. 22	A4988 drivers	4	$1.60	$6.40	https://www.aliexpress.com/item/32965199683.html	
Optional, helps with cable organization	Cable wrapper 2 m	1	$8.89	$8.89	https://www.ebay.com.au/itm/2M-Spiral-Cable-Wrap-Tidy-Cord-Wire-Banding-Storage-Organizer-10mm-25mm-3-Colors/293034936624	Plastic
Connect stepper motors to board	Stepper motor cable extensions	4	$0.40	$1.60	https://www.aliexpress.com/item/32818196194.html	Metal, plastic
Connects board to computer	USB cable, 5 m long	1	$7.95	$7.95	https://www.ebay.com.au/itm/USB-2–0-Type-A-Male-to-B-Printer-Cable-for-HP-Canon-Dell-Brother-Epson-Xerox/111656844488	Metal, plastic
Power supply for control board	24 V, 5A power supply	1	$35.00	$35.00	https://www.ebay.com.au/itm/AU-Power-Supply-Adapter-Transformer-AC240V-To-DC24V-1–2-3–4-5A-for-LED-Strip/202120736297	Metal, plastic
Below: 3D printed items						
Section 3	Linear carriage and half gripper	1	<$0.50	<$0.50	Section 3.3	PLA
Section 3	Stepper motor mount	1	<$0.20	<$0.20	Section 3.3	PLA
Section 3	Half gripper for powder container	1	<$0.05	<$0.05	Section 3.3	PLA
Section 3	Support for forceps	1	<$0.10	<$0.10	Section 3.3	PLA
Section 3	Tray for tin capsules	1	<$1.00	<$1.00	Section 3.3	PLA
Section 3	Tray for powder container and tin capsule	1	<$1.00	<$1.00	Section 3.3	PLA
Section 3	Powder container	1	<$0.05	<$0.05	Section 3.3	PLA
Section 3	Powder container lid	1	<$0.05	<$0.05	Section 3.3	PLA

## Build instructions

### 5.1: Modifying aluminum extrusion profiles

**Fig. 2:**
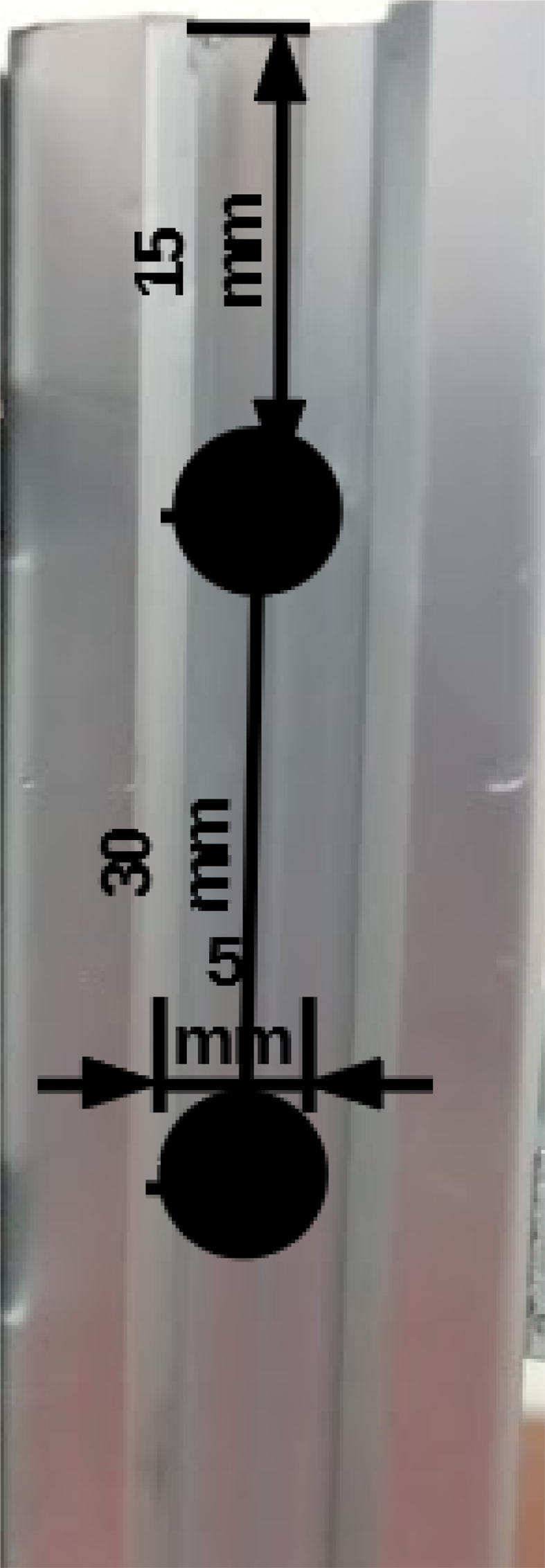
Drilling positions on 250 mm 20 × 20 mm extrusion profile for M5 screws.

Drill 5 mm holes at the positions indicated for one 20 × 20 mm, 250 mm profile (Fig. 2).

**Fig. 3:**
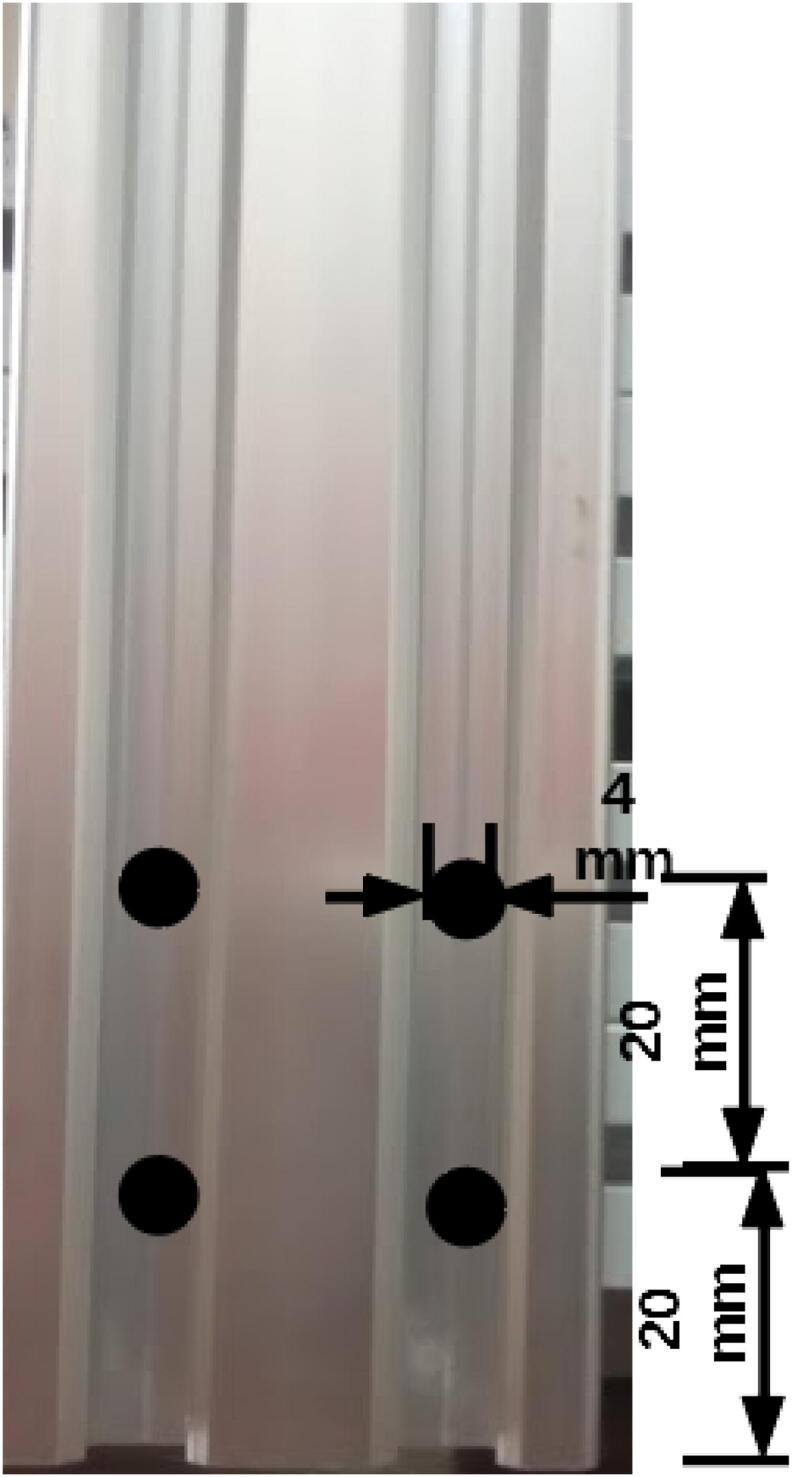
Drilling positions on 500 mm 20 × 40 mm extrusion profile for M4 screws.

Drill 5 mm holes for two of the 20 × 40, 500 mm profiles (Fig. 3).

### Making the base

**Fig. 4:**
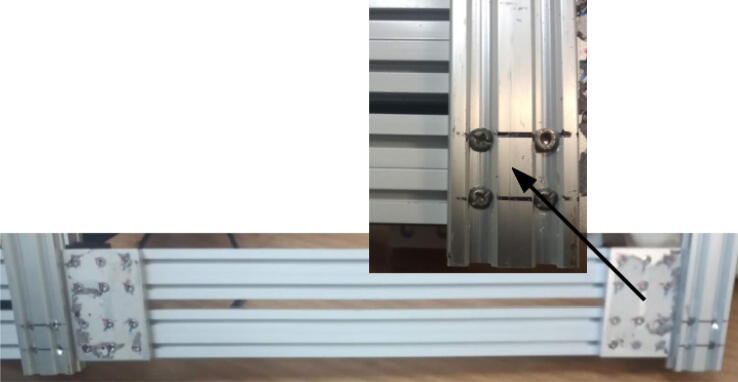
500 mm 20 × 40 mm profiles connected to 700 mm linear actuator.

Connect the drilled 20 × 40 mm, 500 mm extrusion profiles to the 700 mm linear actuator using 20 mm M4 screws through the holes drilled in the previous section (Fig. 4). Do not fully tight the screws at this point, do it just enough for the profiles become united to the actuator.

**Fig. 5:**
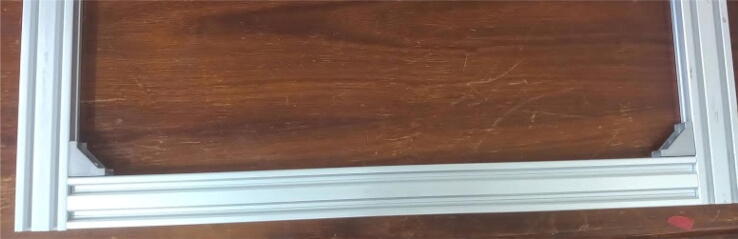
Three 500 mm 20 × 40 mm profiles connected using 2028 corners and M5 screws. The two parallel profiles have the holes drilled as previously described, and are connected to the 700 mm linear actuator.

Connect the remaining 20 × 40 500 mm profile to the other two 20 × 40 500 mm profiles using 2028 corners, and 10 mm M5 screws, spacers and hammer nuts (Fig. 5).

Tighten all screws, including those connected to the 700 mm actuator, and make sure the base is level by placing it on a flat surface and adjusting any screws if necessary.

### Connecting the Y and Z axes

**Fig. 6:**
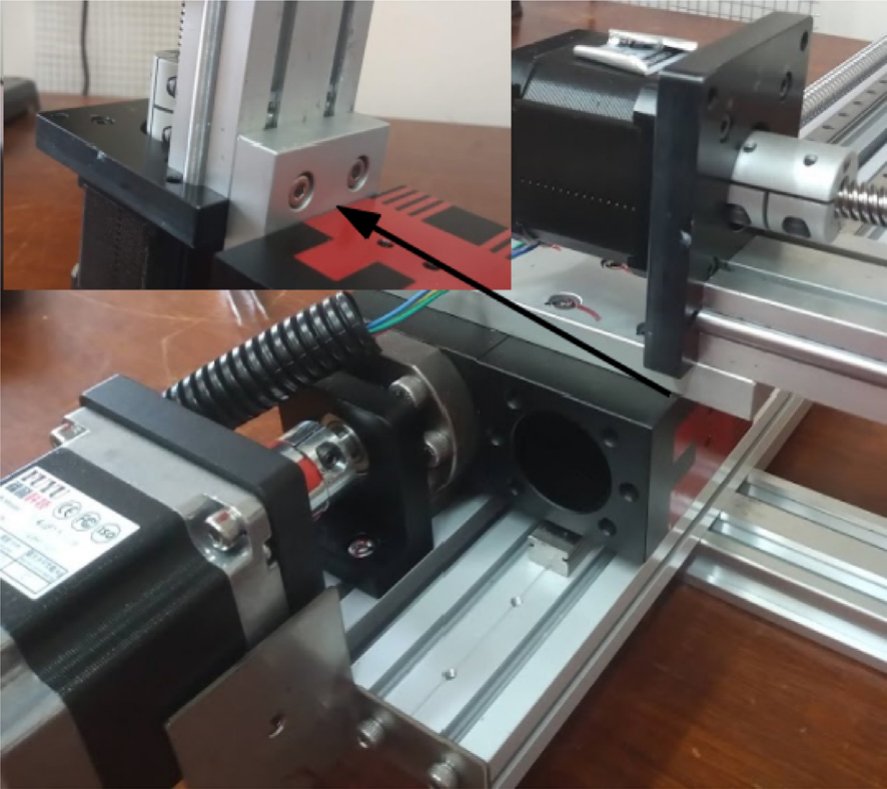
Y axis connected to the X axis. At the top-right corner, see view from beneath, showing the screws connecting the two axes.

Place the Y axis on top of the X axis (700 mm linear actuator) carrier, and attach it using M4, 8 mm screws (Fig. 6).

**Fig. 7:**
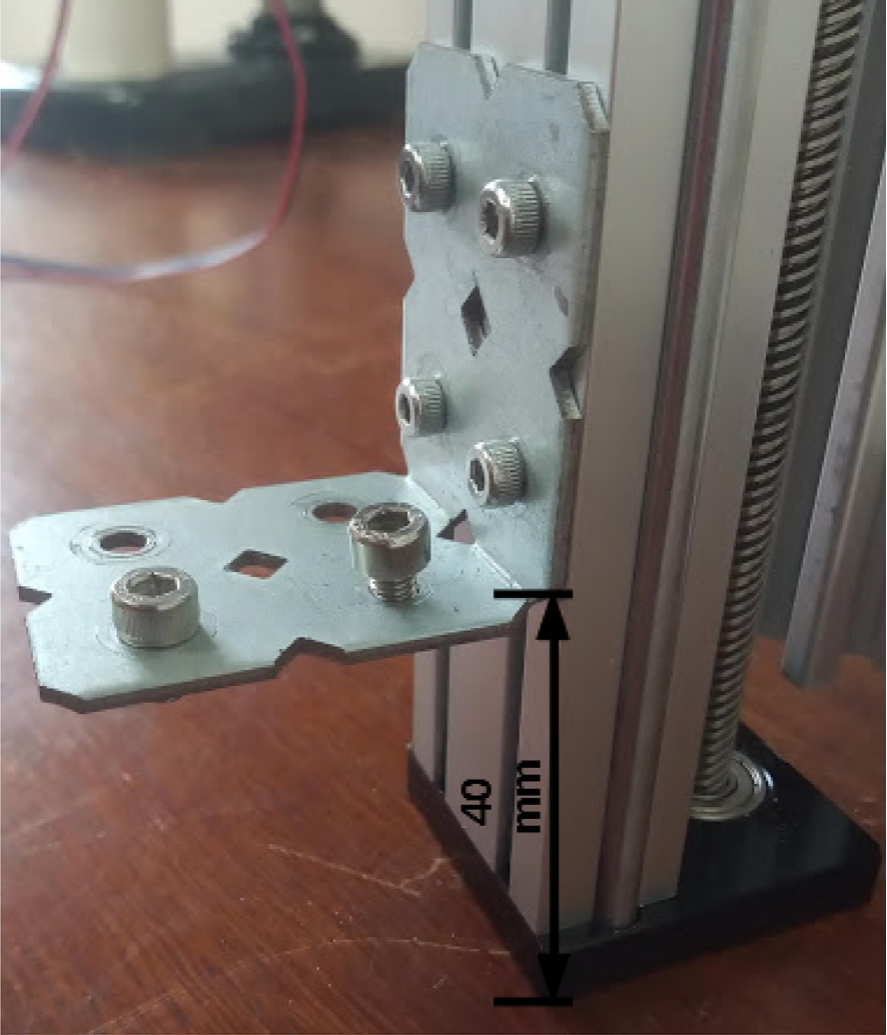
Long bracket connected to Z axis using four 8 mm M4 screws. Two 10 mm M5 screws are positioned horizontally.

Attach a long bracket to the remaining 200 mm linear actuator (Z axis) using 8 mm M4 screws (Fig. 7).

**Fig. 8:**
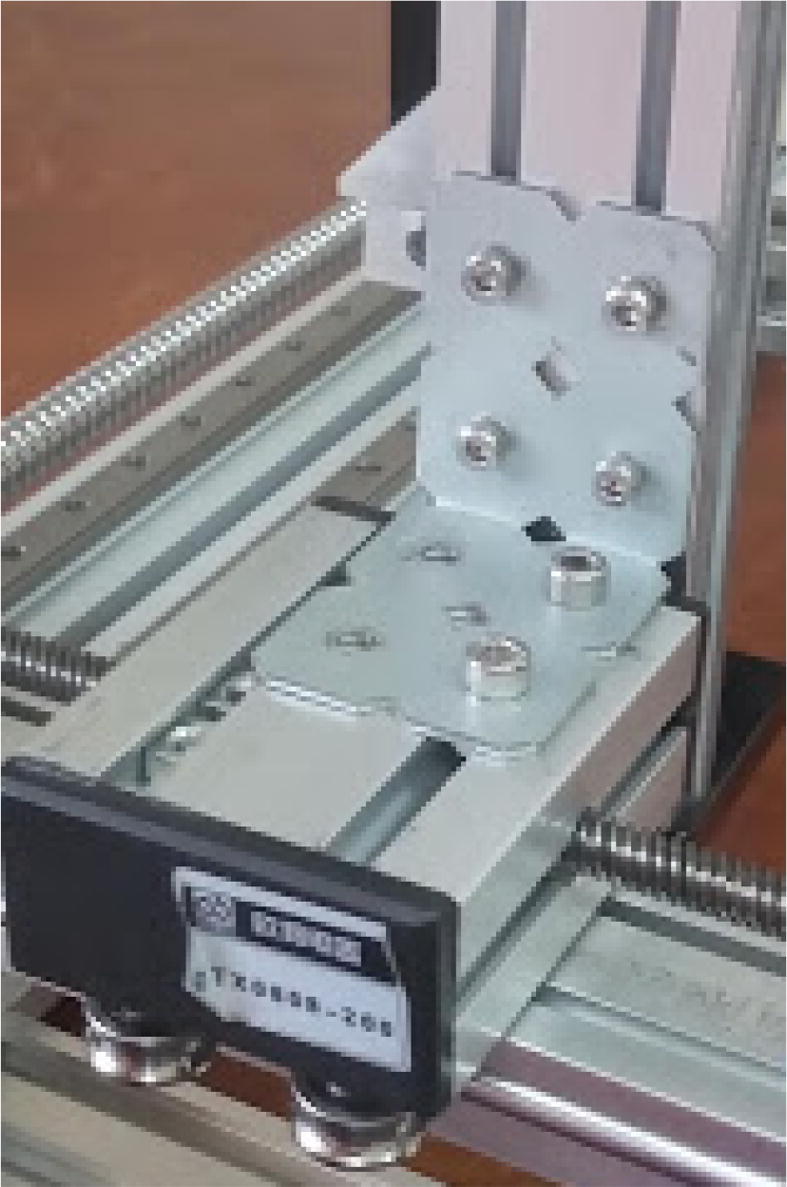
Z axis connected to Y axis carrier.

Connect the Z axis to the Y axis carrier using 10 mm M5 screws (Fig. 8).

### Making the E axis

**Fig. 9:**
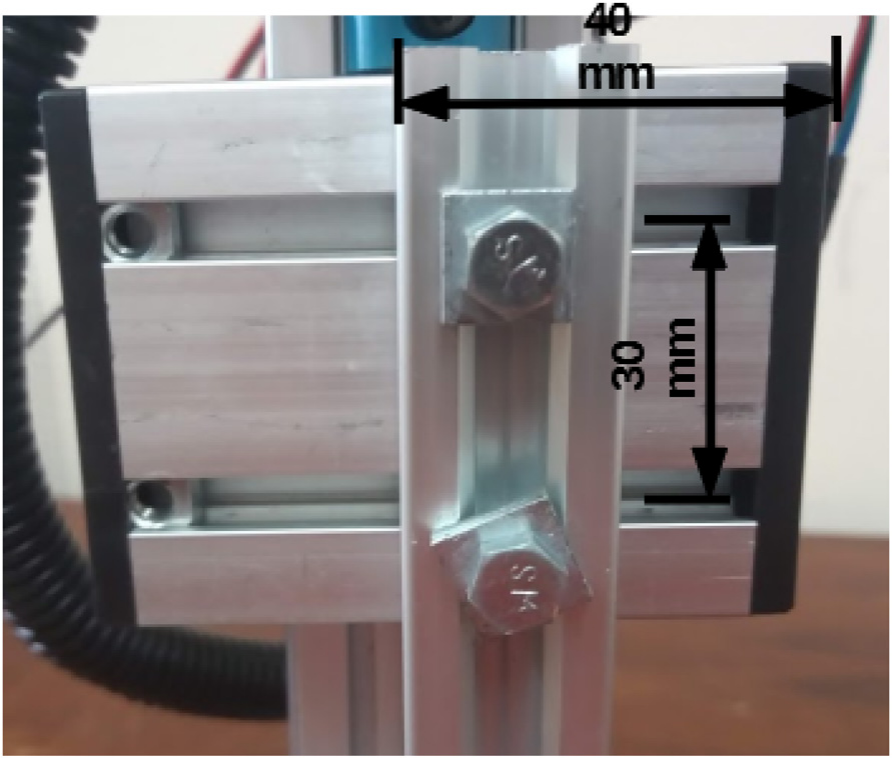
250 mm 20 × 20 mm profile attached to Z axis using 30 mm M5 screws.

Connect the drilled 20 × 20 mm, 250 mm profile to the Z axis carrier using 30 mm M5 screws (Fig. 9). Washers may be necessary to ensure proper attachment of the screws.

**Fig. 10:**
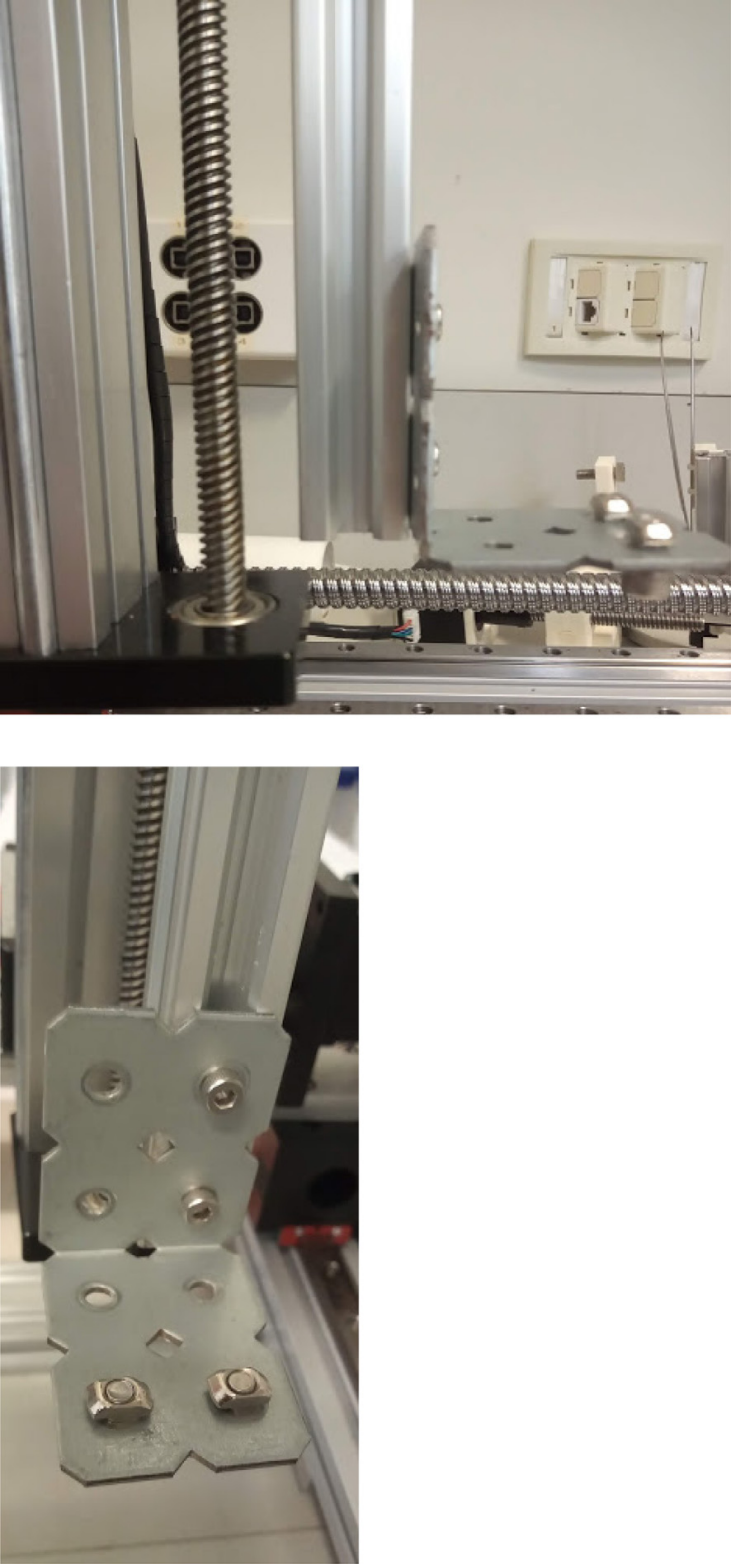
Long bracket attached to the bottom of the 250 mm 20 × 20 mm profile.

Attach a long bracket to the bottom of the 250 mm 20 × 20 mm profile using two M4 8 mm screws and hammer nuts (Fig. 10).

**Fig. 11:**
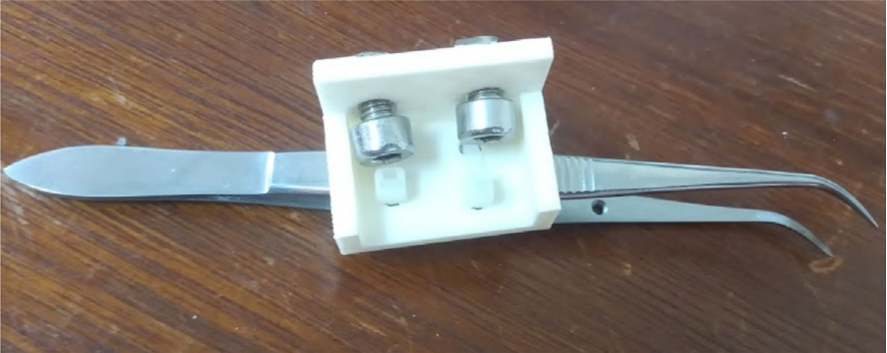
Forceps attached to its holder using cable ties.

Attach the forceps to the forceps holder using cable ties (Fig. 11).

**Fig. 12:**
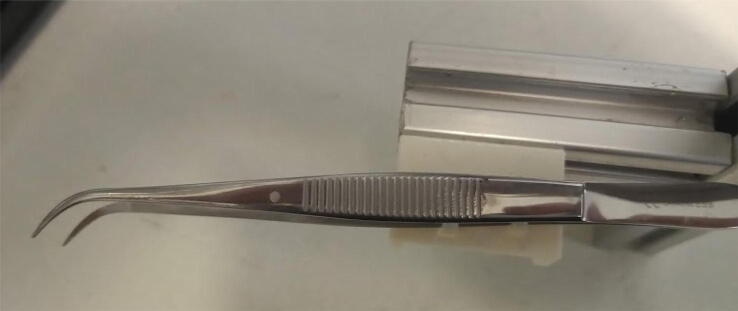
Forceps holder attached to 50 mm profile.

Attach the forceps holder to the 50 mm 20 × 20 mm profile using M5 screws and hammer nuts (Fig. 12). This profile was cut from a 100 mm profile, as 50 mm profiles are not often found for sale.

**Fig. 13:**
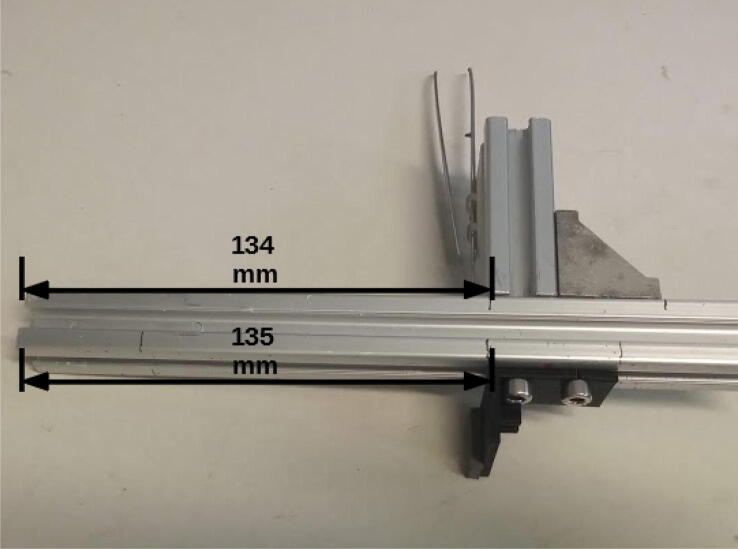
Half gripper and 50 mm 20 × 20 mm profile connected to E axis.

Attach the half gripper for powder container (already with its foam) at a position 135 mm from the profile extremity using M5 screws and nuts (Fig. 13). Attach at the 134 mm position on the opposite side the 50 mm profile with the forceps.

**Fig. 14:**
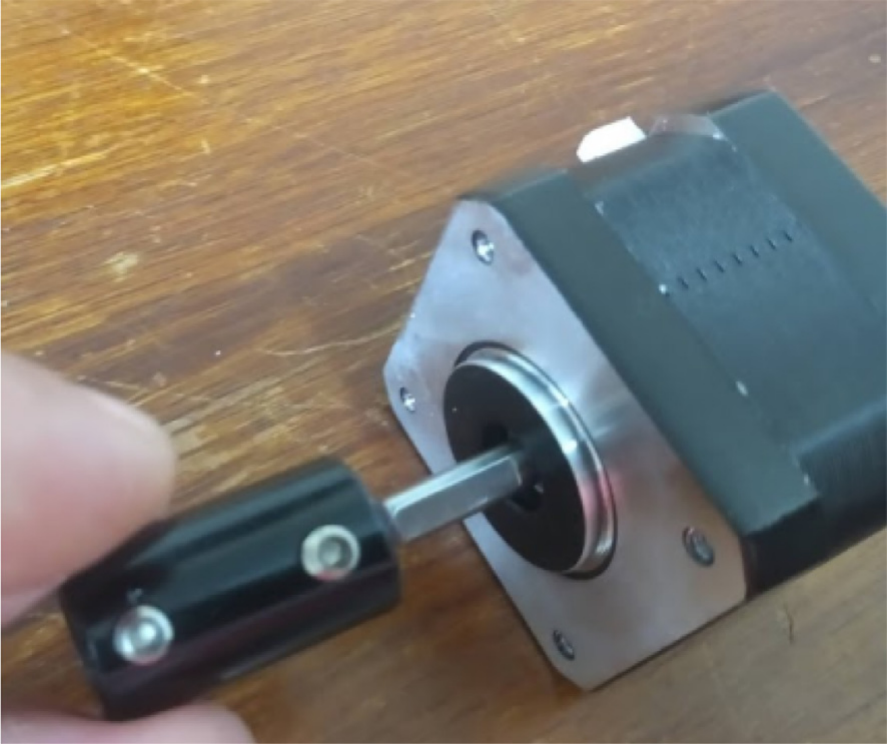
Attaching the shaft connector to the stepper motor.

Attach the shaft connector to the stepper motor (Fig. 14).

**Fig. 15:**
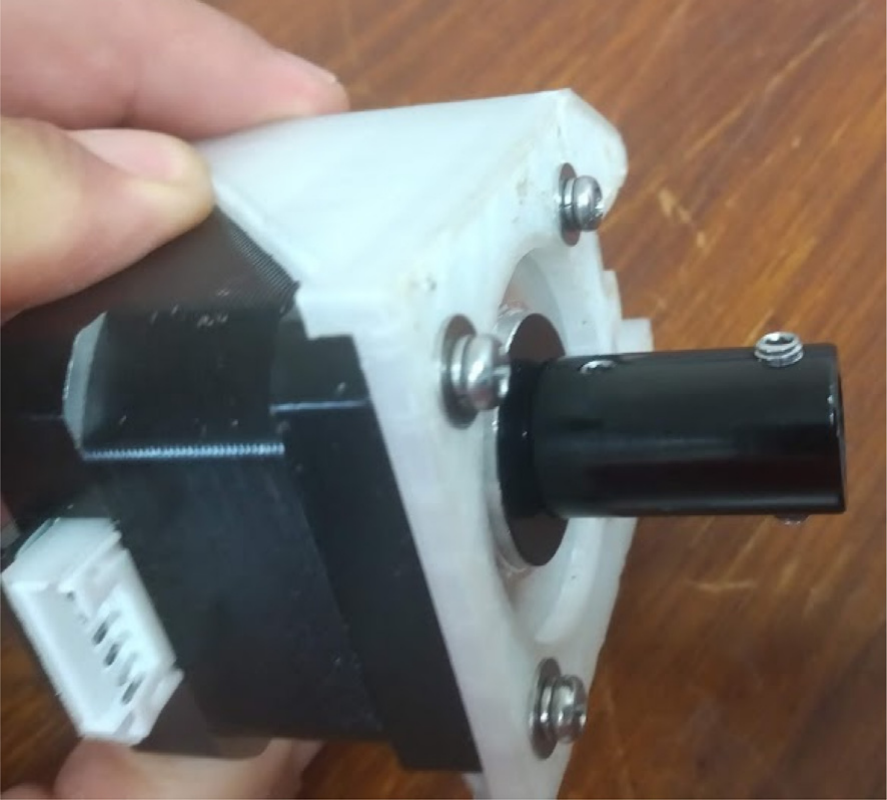
Attaching the stepper motor to its mount using M3 screws.

Attach the stepper motor to its mount using M3 screws (Fig. 15).

**Fig. 16:**
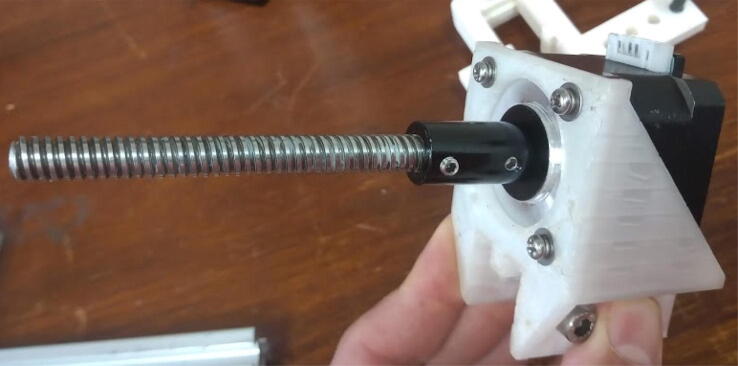
100 mm leadscrew connected to the stepper motor using the shaft connector.

**Fig. 17:**
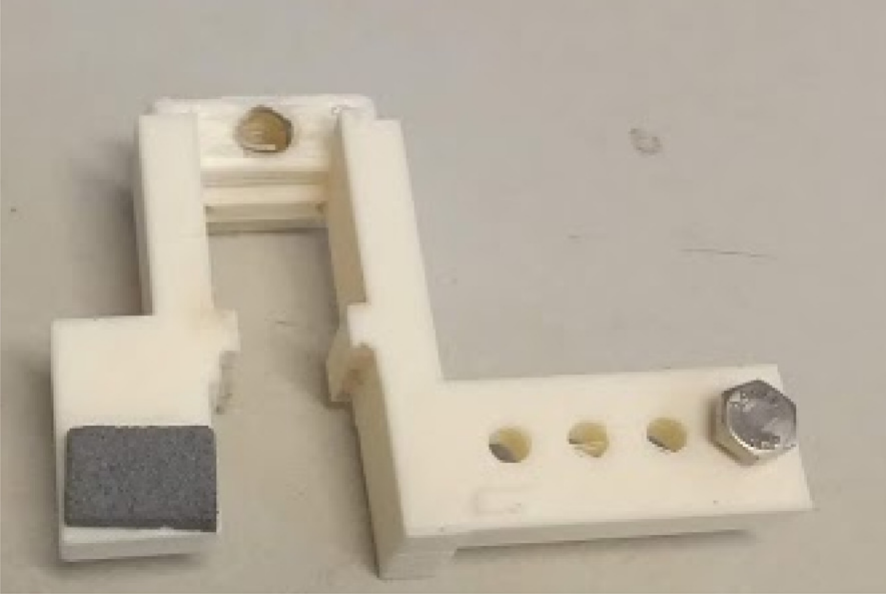
Linear carriage / gripper with foam and M6 screw.

Connect the 100 mm leadscrew to the shaft connector (Fig. 16):

Attach a foam and a M6 20 mm screw to the linear carriage / gripper (Fig. 17).

**Fig. 18:**
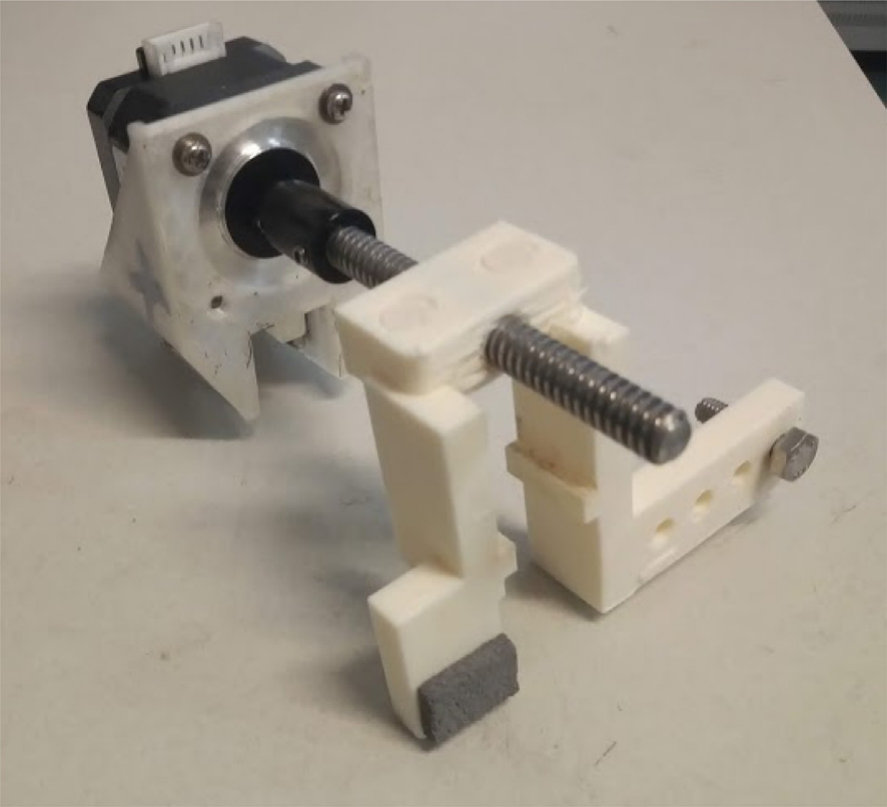
Linear carriage / gripper connected to leadscrew.

Place the linear carriage / gripper around the 10 cm leadscrew (Fig. 18). If it is stiff, make it slide around the screw a few times, and lubricate using grease.

**Fig. 19:**
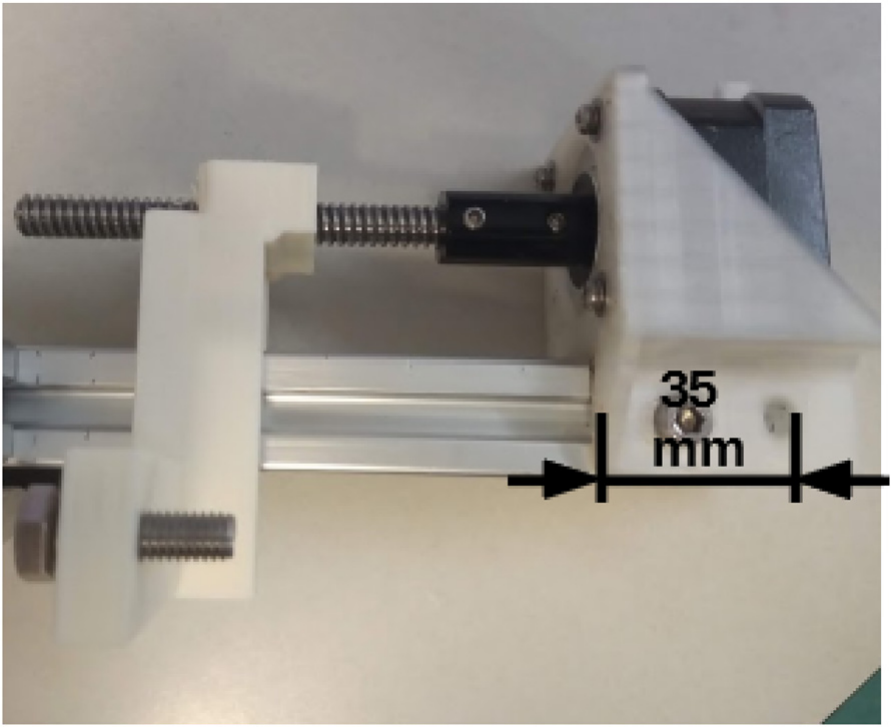
Stepper motor attached to the 200 mm, 20 × 20 mm profile (E axis).

Attach the mount to the 20 × 20 mm, 200 mm profile using 10 mm M5 screws and hammer nuts (Fig. 19). Make sure the face of the motor is 35 mm distant from the extremity of the aluminum profile. Slide the linear carriage / gripper in the grooves of the profile. Apply grease if stiff.

**Fig. 20:**
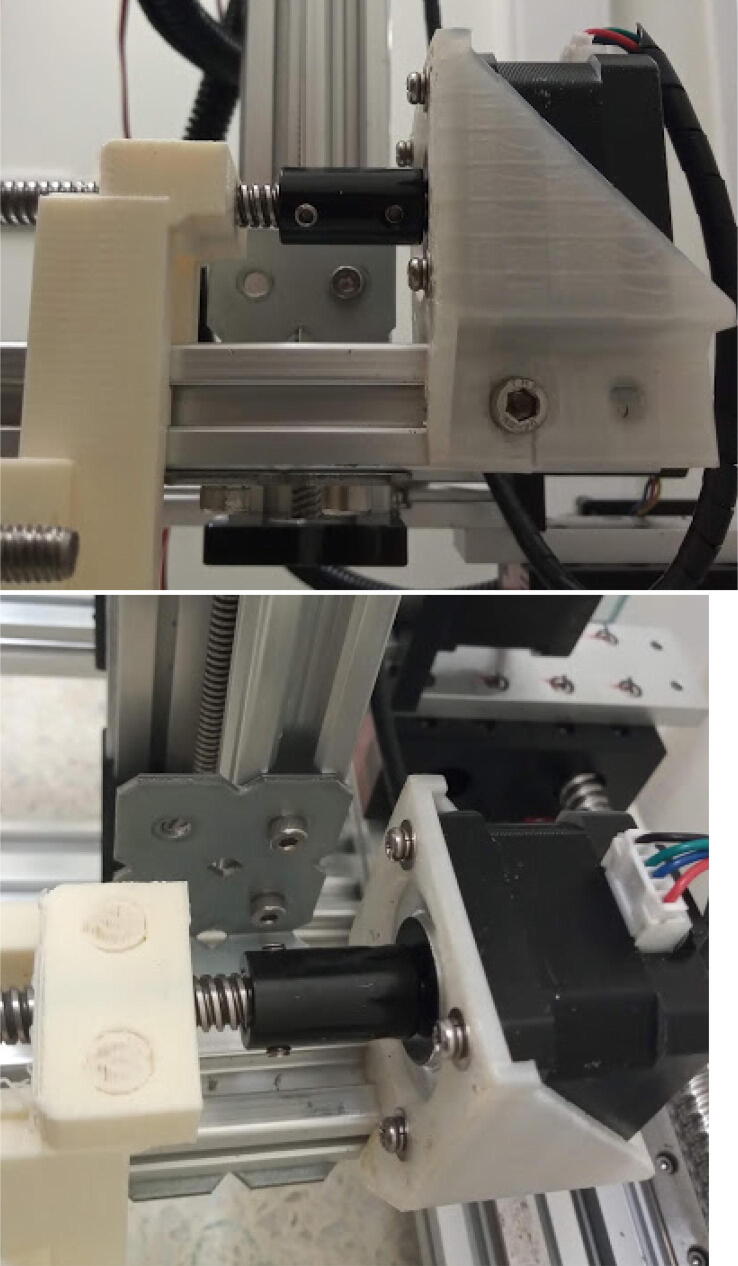
E axis connected to 250 mm 20 × 20 mm profile.

Connect the E axis to the 250 mm 20 × 20 mm profile using the long bracket (Fig. 20).

### Attaching the trays to the base

**Fig. 21:**
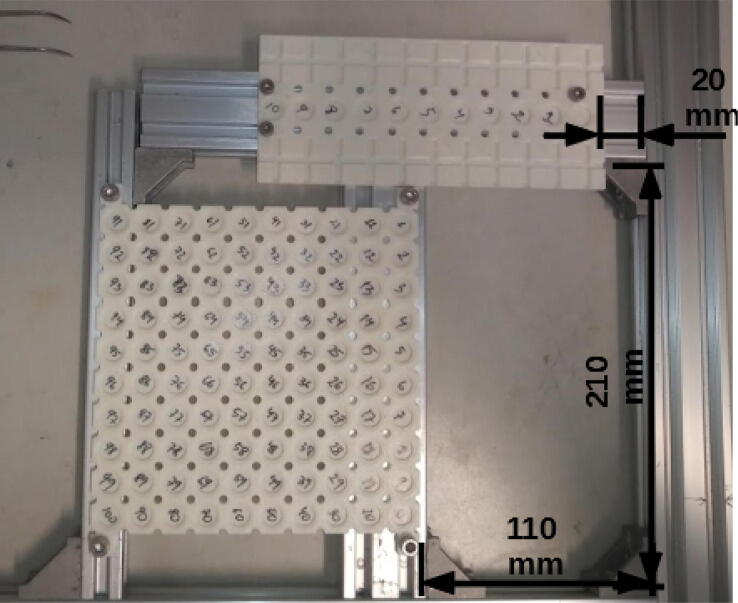
Trays attached to the base. Notice that 20x20 and 20x40 extrusion profiles were connected to the base to provide support for the trays.

The trays are attached using M5 screws (for the tin tray) and M4 screws (deposit and powder tray) and hammer nuts for both cases (Fig. 21).

The tin tray is placed on a 20 × 40 mm, 200 mm profile, and on a 20 × 20 mm, 250 mm profile. The deposit and powder tray is placed on a 20 × 40 mm, 250 mm extrusion profile. The position of the deposit tray on the Y axis is related to the position of the gripper on the Z axis. When operating miau (section 6), test if when holding the powder deposit on the tray the Y position is Z for the gripper. If not, adjust the gripper position in Fig. 20 (the connection between Z and E axes).

### Preparing the control board

Now miau is mostly built, and only the electronic parts need to be prepared. The first step is to prepare the control board. Here, I use MKSgen-L, a board developed for 3D printers. It comes without a firmware, which needs to be uploaded to the board.

The first step is to install the driver for the board. As I use the Windows operating system, the driver is CH340, which can be downloaded from https://sparks.gogo.co.nz/assets/_site_/downloads/CH34x_Install_Windows_v3_4.zip. Install the driver on the computer, and then connect the board to it. The computer should recognize the board.

Once the board is recognized by the computer, the firmware can be uploaded to it. The procedure consists of 3 steps: 1) download and install Arduino, available at https://www.arduino.cc/en/software; 2) download the modified Marlin package available at https://osf.io/y2hu8/ or https://doi.org/10.17605/OSF.IO/Y2HU8; 3) open the package using Arduino, select the board (find the COM port number using Device manager, for example), and upload the package to the board. The Marlin package used here is similar to the ones used in previous devices [8], [9], [10], [22], the modifications being in the federate (maximum and default set as 2000) and acceleration (maximum and default set as 50).

**Fig. 22:**
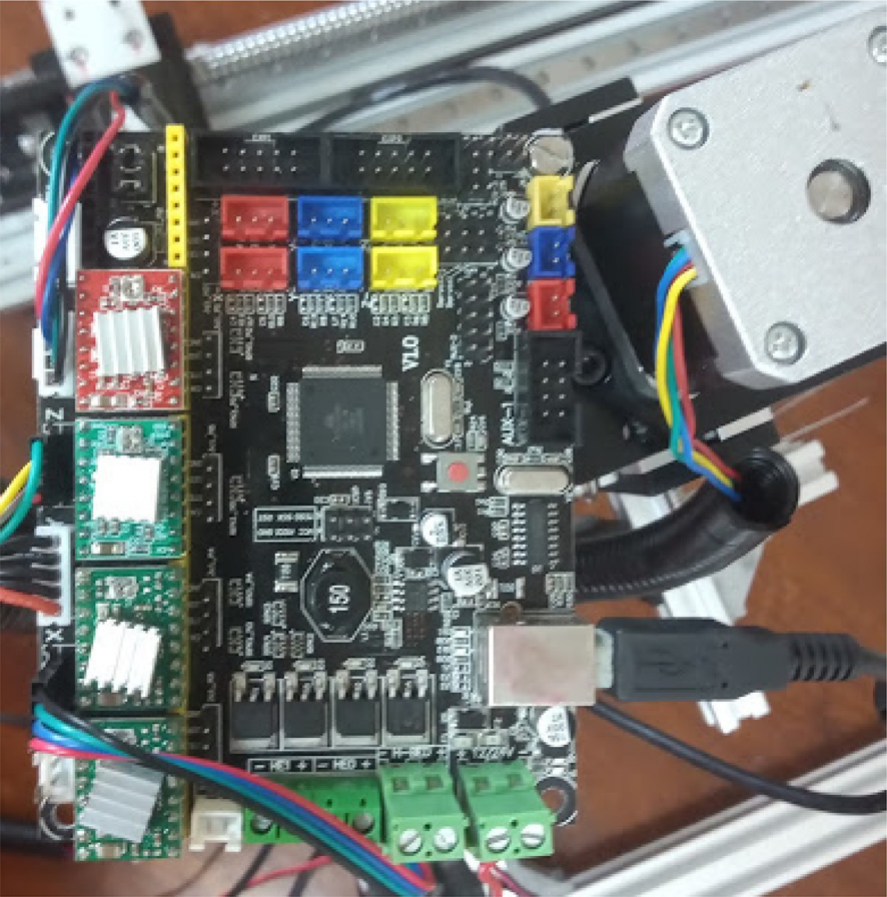
MKSgen-L control board placed at the top of Z axis. Notice the stepper motor drivers with their potentiometers (small screws at one extremity of the drivers) facing away from the side of the board with the power supply cable (bottom of the photo). This orientation must be followed or the drivers can be irreversibly damaged.

Once the firmware is installed, the board can be used to control the stepper motors. Stepper motor drivers need to be attached to the board. Pay attention to the correct orientation when doing that (Fig. 22). The drivers used here are all A4988.

Place the board on top of the Z axis and fix it there using a M4 screw (Fig. 22). Connect the cables from the motors to the control board, each to its respective axis. It may be necessary to employ cable extensions from the motors to the board. Covering the cables with cable protectors can also be helpful. Connect the power supply and USB cables to the control board.

### Arranging miau and the balance

**Fig. 23:**
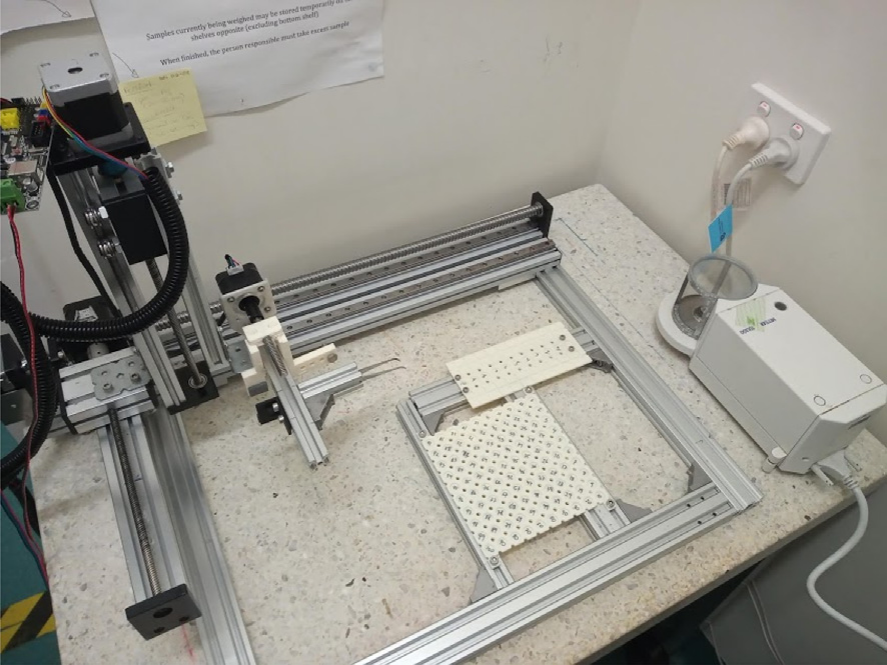
Miau positioned so that the balance dish is inside reach of the forceps.

Arrange the miau and the balance in such a way that it is possible for the forceps to reach inside the balance chamber (Fig. 23). Remember to adjust the balance inclination after moving it (in most balances, this is done using a bubble) to obtain accurate masses.

### Powder container

**Fig. 24:**
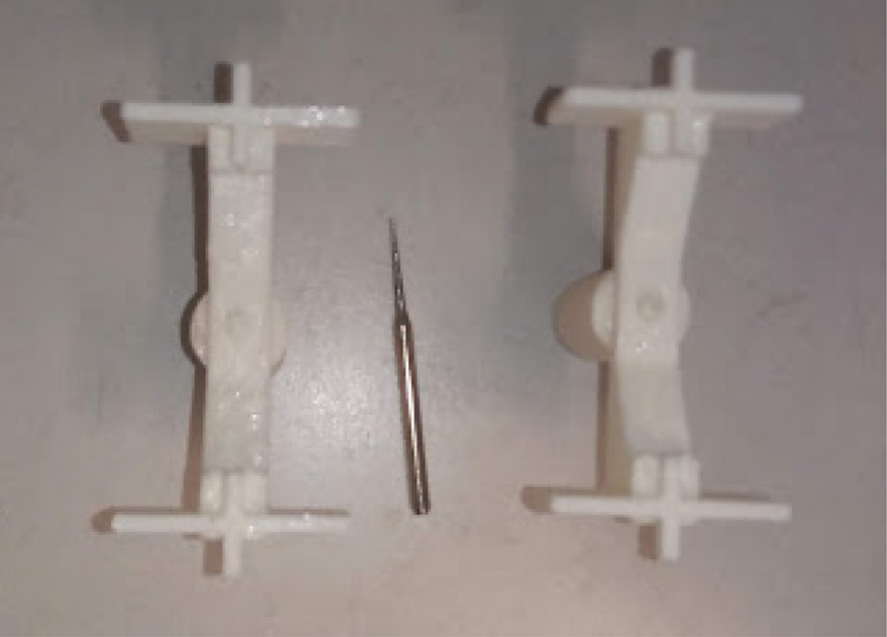
Powder container. The one at the left has not a hole, the one to the right has a hole made with the drill (about 0.5 mm in diameter) at the middle.

The powder container needs to be drilled with a fine drill to make a hole of about 0.5 mm. Drills for routers (for example, Fig. 24) have fine tips and do not brake easily. The actual hole size depends on the powder being delivered. This hole size worked for powders with particles sieved using a 0.5 mm mesh.

## Operation instructions

### Motor and balance control

Motor control is done using G-code [28] via the program Hype!terminal (available from https://osf.io/y2hu8/ or https://doi.org/10.17605/OSF.IO/Y2HU8). The serial connection settings are: 115,200 for Baud rate, 8 data bits, none for parity, 1 stop bit, and none for flow control. The G-code dialect used is that for Marlin, thus including M commands [8]. The stepper motors are controlled using the G1 command: G1 < axis><distance><speed><movement speed>. For example, the command G1X10F500 means that the motor controlling the X axis will move 10 steps at 500 steps per minute. Important: before starting sending G commands, it is necessary that the command M121 is sent (once every time connection between board and computer is stablished), so that the full range of step values (including negative ones) can be reached.

The balance used here allows control using a computer. For this, a serial cable must be connected between the balance and the computer. Commands to operate the balance are available in its manual. Commands were sent to the balance using AutoIt. The library Commg.au3 and the file Commg.dll were used, and are available at https://osf.io/y2hu8/ or https://doi.org/10.17605/OSF.IO/Y2HU8.

### Weighing algorithm

**Fig. 25:**
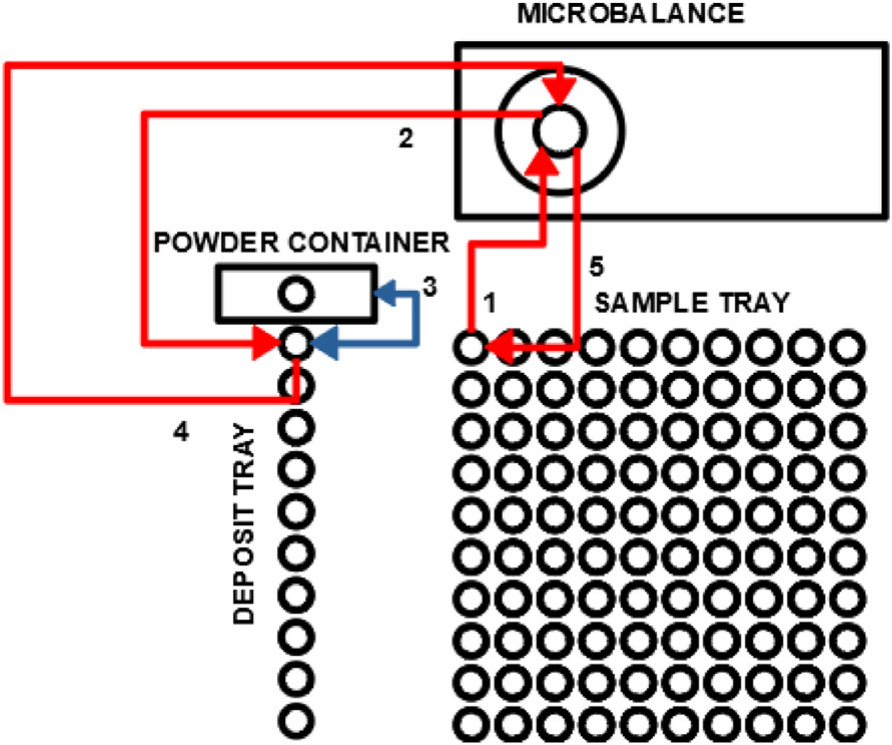
Positions and movements in the weighing procedure. Red arrows show tin movements. Blue arrow shows powder container movements. Numbers besides the arrows show the sequence of actions. This sample tray has 100 positions. The movements from and to it can be from any of these positions. The deposit tray has 10 positions. The movements are usually only for the first (topmost, in the figure) position, but change if problems arise. If all deposit positions get used and another problem arises, the run is cancelled. (For interpretation of the references to colour in this figure legend, the reader is referred to the web version of this article.)

**Fig. 26:**
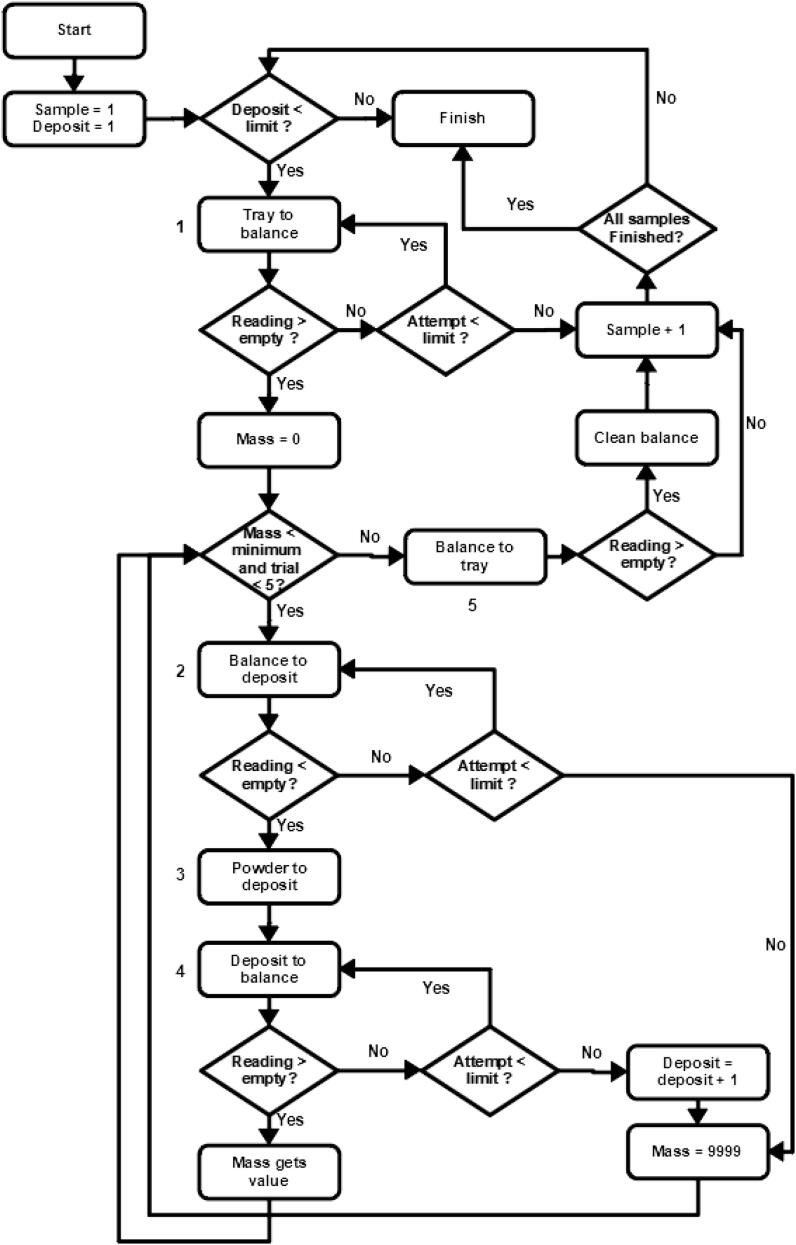
Weighing flowchart. Numbers besides boxes indicate steps in Fig. 25.

The weighing procedure consists in a series of synchronized actions between miau and the balance (Fig. 25, Fig. 26). This ensures that the procedure is done in a closed loop, that is, actions are not performed blindly [29]. The feedback is provided by the balance by checking the present mass at key steps.

The software controlling miau and the balance attributes the value of 1 to two variables in the code, “deposit” and “sample” (Fig. 26, just after start). This means that the positions to be reached at the deposit and sample trays are positions 1. For the deposit tray, this is the topmost position in Fig. 25. For the tin tray, this is the position at the top left corner. Deposit position 2 is the next one from top to bottom. Sample positions increase from left to right, then return to left when the line is finished, and go down one line.

Miau picks an empty tin capsule and takes it to the balance (step 1, Fig. 25). The balance checks the mass. If the value is less than an empty balance (this limit is set in the program code), then miau returns to the tray and repeats step 1 again (Fig. 26). Miau will try 5 times, with small differences in the positioning every 2 times (this level of detail is not shown in Fig. 25 or 26). If miau fails all the times, it goes to the next sample (Fig. 26). If it succeeds, it means that the sample is now on the balance dish.

At step 2 (Fig. 25), the balance checks the mass again (Fig. 26): if it is larger than that expected for an empty balance, it repeats the procedure, as this means that the tin capsule is still on the balance dish. As for step one, it tries 6 times. If it fails the 6 times, it will try to remove the tin capsule from the balance dish (clean balance in Fig. 26), and move on to the next sample. If it succeeds, it means that the tin capsule is now at the deposit.

Before step 3, the program attributes the value 0 to the variable mass in the program (Fig. 26). Then there is a check: if mass is larger than a set value, miau will perform the final steps of the procedure. As mass now is zero, and the set value must be higher than that (this is the minimum value for a useful sample; in our laboratory, 0.3 mg), the procedure follows the yes route (Fig. 26, beneath “mass < minimum?” field). Thus, the next step will be to transfer powder to the tin capsule in the deposit (step 3, Fig. 25). Notice that this is a back and forth movement, as the deposit returns to its original position when finished. Now it is time for step 4, that is, the transfer of the tin capsule from the deposit to the balance (Fig. 25). Again, there is a check (Fig. 26). If the balance is empty, meaning that the tin is still at the deposit, miau will try again, 5 times in total. If it fails all times, the variable deposit will be incremented by 1, and miau will move on to the next sample. From now on, all next samples will be transferred to position 2 in the deposit tray. If the same problem happens repeatedly, to the point that after position 10 in the tray is reached it happens again, the sequence is cancelled (all these actions are shown in Fig. 26). If it succeeds, it means that the tin is now on the balance dish. There is another check, then: if the current reading minus that of the empty tin (obtained in step 1) is less than the set value, the entire procedure listed in this paragraph is repeated. This is a potentially infinite loop, and as many trials can be attempted as needed until the minimum mass is achieved. This can literally take forever, so a limit is also stablished, and only five trials are allowed (Fig. 26).

Once a tin capsule holding at least the minimum amount of sample is on the balance dish, miau performs step 5 (Fig. 25), which is simply returning the tin to the sample tray. As for all previous steps, there is a check here (Fig. 26), and if the balance is not empty, miau will try 5 times. If it fails, it will clean the balance and move to the next sample (this is not shown in Fig. 25, only in Fig. 26). If it succeeds, it means that the tin capsule is back at the tray (or at least not in the balance anymore) and miau moves on to the next sample.

Some movement details are not described in the general algorithm, and will be briefly mentioned only: 1) While it is possible to perform horizontal movements on the two axes (X and Y) simultaneously, it is not advisable to perform simultaneous vertical and horizontal movements. This is important to avoid collisions. Other movements are also designed to avoid collisions due to several factors. 2) Manipulation of tin capsules often results in their deformation. The main consequence is that movements must be carefully designed to avoid or adapt to these deformations. For example, tin capsules are taken from the tray at a certain vertical position, but are displaced back at a higher position. This is difference avoids the tin capsule to become squashed when being put back.

### Autosampler operation

**Fig. 27:**
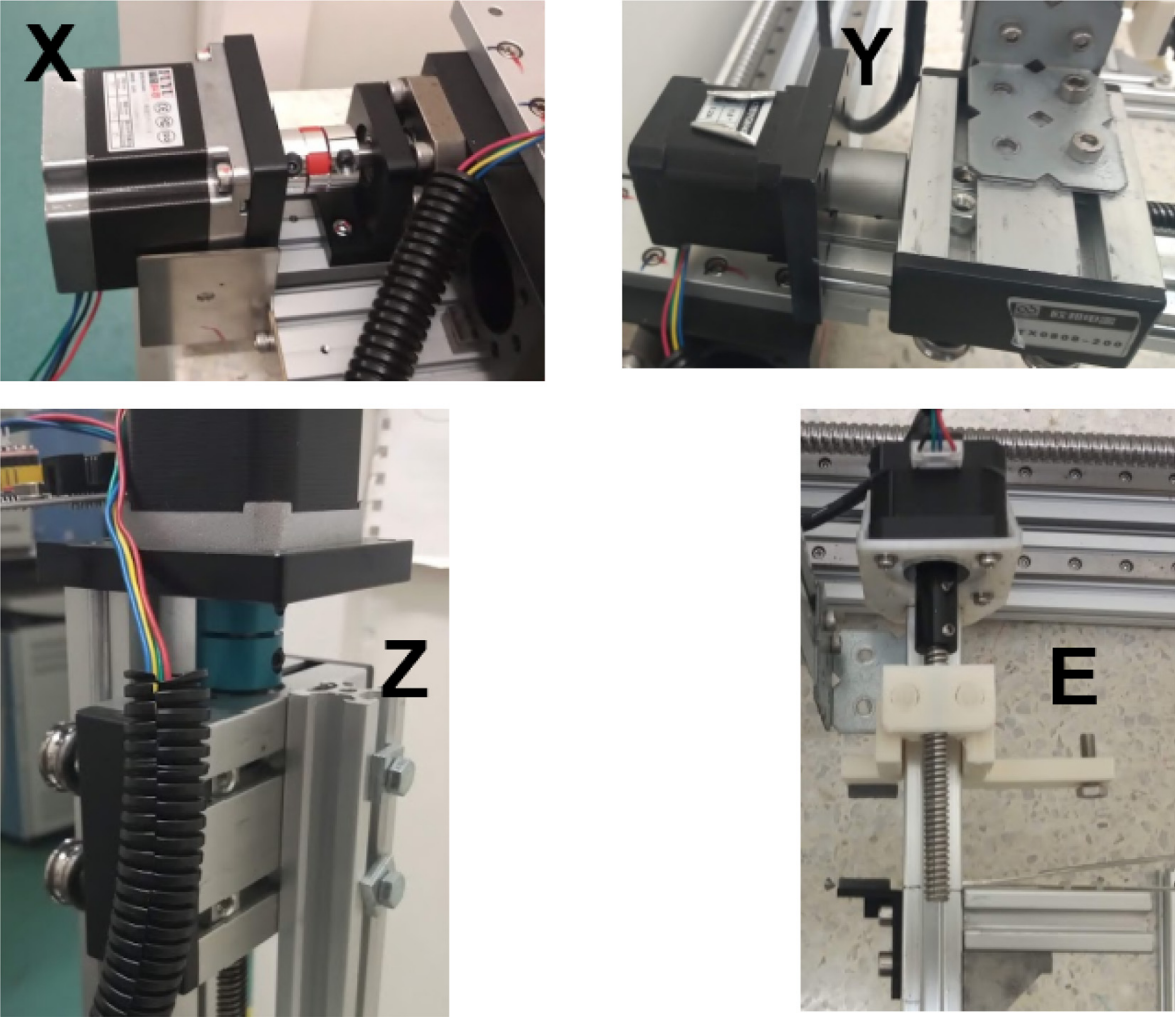
Start (zero) positions for the four axes.

Before using the script and operating miau with the balance, it is necessary that miau has all axes at start positions, that is, zero positions in the axis coordinates (Fig. 27).

All actions described in the previous section are performed by miau (tin capsule and powder container manipulation) and the balance (weighing). Both instruments are controlled using the computer, and synchronized using AutoIt (the full script is explained in detail in Supplementary information 2). Both miau and balance must be connected to the computer using the appropriate cables. Hype!terminal must be connected to miau control board, and the COM port number for the balance must be known and listed in the AutoIt script. All positions in the script must be verified before first use; the numbers provided here (supplementary information 2) are strictly for the machine built in our lab. It is likely the values will be different for different machines. Once these positions are adjusted (by manual trial runs), the script can be run. With the design provided here, up to 100 samples can be processed in a single unattended run.

## Validation and characterization

### Powder selection

I have not performed a comprehensive evaluation of the powders suitable to be delivered using the powder container presented here. So far, I have observed that the mechanism works well for powders with grain size less than 0.5 mm in diameter (which can easily be achieved by means of sieving). Also, it works best with powders that do not tend make “lumps” of smaller grains. Because of that, it is important to use only fresh, dry powder that has been stored in sealed containers, as opposed to powders that have been sitting in a humid room for example.

As a recommendation, substances provided by popular chemical suppliers, which are often used as working standards for isotope and elemental measurements, work well after sieving. I have tested glycine (G7126, Sigma Aldrich), glucose (G8270, Sigma Aldrich) and rhamnose (W373011, Sigma Aldrich). After sieving, they all work well.

### Weighing precision and accuracy

The main criteria to determine whether miau performs a useful job are weighing precision and accuracy. The concern is that the automated procedure could add uncertainty to the weighing procedure. This could happen for a number of reasons, including: 1) loss of powder when placing the tin capsule on the balance dish; 2) loss of powder when transferring the tin from the balance dish to the tray; 3) intrusion of different powders when the tin capsule is left sitting on the tray; 4) damage of tin capsule resulting in change of its mass.

Visual inspection did not show evidence for any of such problems, but a verification was made by comparing performing the weighing of glycine powder both manually and automatically using miau. A total of 40 tin capsules were placed on the sample tray of miau, and it was put to work. The powder was glycine (G7126, Sigma Aldrich, previously sieved at 500 µm). In the end, 38 full capsules were obtained (a loss of 5%, common in the usage of miau), with masses between 0.3 and 1.1 mg. Another 40 samples in a similar mass range were prepared, but manually. All samples were measured for N content and isotope composition using an elemental analyzer (Flash EA, Thermo Fisher), equipped with an autosampler [10] and coupled to an isotope ratio mass spectrometer (Delta V plus, Thermo Fisher). Automated and manual samples were run at alternate batches of 10 each to avoid possible drift in the measurements.

**Fig. 28:**
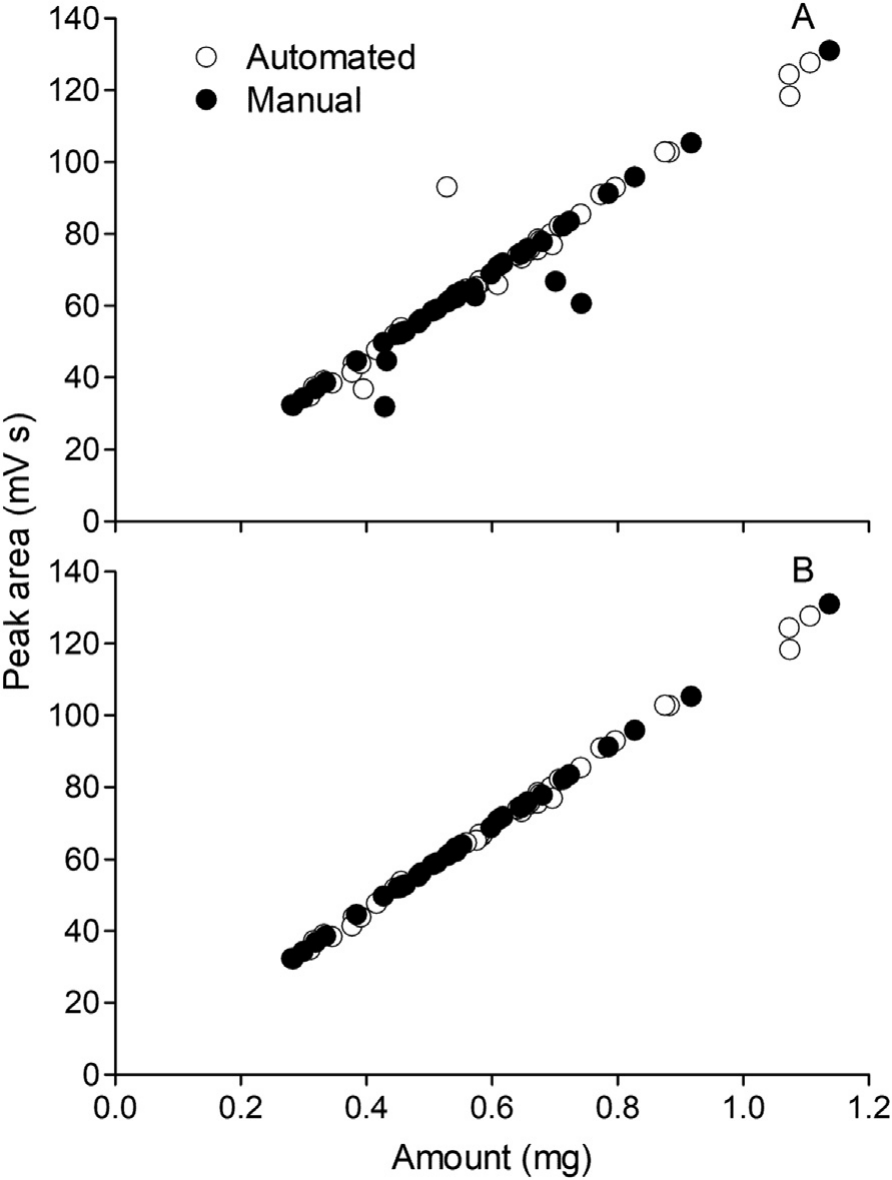
Peak area obtained for different amounts of glycine prepared either automatically or manually. A: all samples included. B: outliers due to handling error removed.

There was error in the measurements, as evidenced by points deviating from the main regression line (Fig. 28A). These were almost certainly caused by errors when wrapping the samples after weighing, and were more common for manual than for automated samples. To determine if automated and manual weighing performed equally, the ratio between N content in glycine and the obtained peak area was compared for the two data sets. When doing this comparison, it was found that there were 3 outliers for automated samples, and 5 for manual samples (Turkey Fence method, k = 1.5). Removing these outliers, the regression lines became almost undistinguishable (Fig. 28B), indicating that both procedures performed similarly. Finally, a Student *t* test showed that the ratio between peak areas and N content for the samples (without the outliers) were not significantly different for the two treatments regarding the means (automated: 1.624 ± 0.032, n = 35; manual: 1.617 ± 0.010, n = 35; p = 0.199). This shows that the procedure was accurate. It also shows it was precise, as the error in the measurements was similar for both automated and manual procedures.

Isotope measurements were also undistinguishable between the two data sets (automated: 2.0 ± 0.10, n = 38; manual: 2.0 ± 0.07, n = 40; p = 0.143; Student *t* test for means). These results are in accordance with the precision expected for this measurement [4]. Therefore, the test demonstrates that the automated procedure is as good as the manual one for this kind of analysis.

### Efficiency and usefulness

The setup of miau for initial use is simple: the balance needs to be repositioned from its position for manual weighing, and miau placed on the sampling table. The computer setup is very simple and straightforward using the script provided here (available at https://osf.io/y2hu8/ or https://doi.org/10.17605/OSF.IO/Y2HU8; explained in Supplementary Information 2). Therefore, the balance can be easily used automatically or manually in accordance with the purpose. The fact that AutoIt is used to integrate miau to the balance means that any other balance controlled by a computer can be used with miau. This makes miau readily available to a large number of laboratories equipped with microbalances and performing stable isotope analyses.

Miau is not particularly fast in its operation, typically taking about 5 min to weigh one sample. This performance is comparable to that by machines in the order of tens or hundreds of thousands of dollars (3 to 5 min per sample [1]). Manual weighing can be faster (typically 1 to 2 min for a well-trained person; it largely depends on the time the balance takes stabilizing the mass reading). However, using miau, the time a person takes dealing with the balance is only 5 s, which are used to place the tin capsule in the tray (wrapping the sample afterwards still needs to be done, but this needs to be done manually as well). Therefore, the automated procedure results in the reduction of more than 90% in the time dealing with these samples.

When measuring stable isotopes in solid samples, it is good practice to intercalate samples with standards for quality control monitoring [4], [5]. Typically, 1 to 3 standards are placed every 10 to 20 samples in a sequence to monitor for drifts. Thus, in a typical run between 10 and 30% of the measurements are for standards. Since miau is best suited for standard preparation, it can be argued that using it can alleviate ~20% of the sample preparation work for stable isotope measurements.

Finally, people measuring stable isotopes in solid samples are probably questioning why not employ miau to automate sample weighing, in addition to standard weighing, as proposed here. The answer is that difficulties arise when dealing with more than one substance with miau. There is the obvious possibility of cross contamination between samples. However, the largest obstacles are 1) dealing with a multitude of different materials, which will each demand a different container hole size / dropping cycle / delivery mass (some substances need around 1 mg for analysis, others around 5 mg, others 50 mg, etc.); and 2) using hundreds of containers, one for a different sample. When dealing with a single substance many times, there is only one container, while when dealing with multiple samples there is need of a container for each sample. Even if the containers could be purchased by a supplier (currently they need to be 3D printed), the work involved in storing the samples in the containers, testing them to determine the right pore size, etc, would most likely consume equivalent or even more time than simply weighing the sample directly in the balance. Thus, I cannot recommend miau for the task of weighing many different samples in a single run. Of course, it is possible that a different approach based on different powder transfer be more suitable for this task [3].

## Declaration of Competing Interest

The authors declare that they have no known competing financial interests or personal relationships that could have appeared to influence the work reported in this paper.
