# Supplementary material for: Miau, a microbalance autosampler
Source: HardwareX. 2021 Jun 25;10:e00215. doi: 10.1016/j.ohx.2021.e00215 (PMC9123439; doi:10.1016/j.ohx.2021.e00215)

**Explanation for AutoIt code**

For a full understanding of the code described here, it is advantageous for the reader to study the book Practical Laboratory Automation Made Easy with AutoIt, by Matheus Carvalho, 2016, Wiley VCH, ISBN: 9783527341580.

**Preparation**

The reader should have the AutoIt editor installed on a Windows computer. The installation files for this program can be downloaded from https://www.autoitscript.com/site/autoit/downloads/.

**Necessary files:**

Commg.dll

Commg.au3

ManuscriptCode.au3

Get the files from the repository at https://osf.io/y2hu8/ or https://doi.org/10.17605/OSF.IO/Y2HU8 . You can use SciTE to open the au3 files. Only ManuscriptCode.au3 will be explained here. Commg.au3 is described in the book Practical Laboratory Automation Made Easy with AutoIt, by Matheus Carvalho, 2016, Wiley VCH, ISBN: 9783527341580.

**Code explanation:**

First lines: configuring the environment


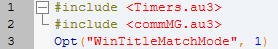


The first 2 lines include 2 libraries necessary for the code. The next line has the Opt command set to make the first part of a window name as the input for commands dealing with windows.

Line 5 declares the variable $Hypecontrol.


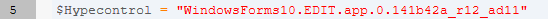


The value for $Hypecrontrol is obtained using the AutoIt Windows Info tool by placing the cursor on the input field on the Hype!terminal interface:


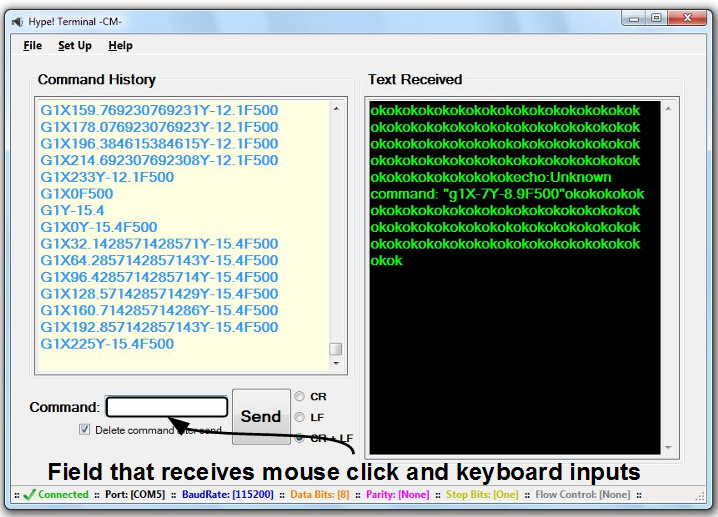


Lines 7 to 10 declare the variables and call one of the functions used to the control of the microbalance through the comport.


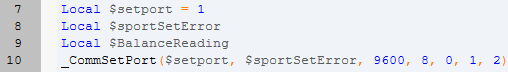


Lines 13 to 64 declare the variables that determine important positions for the movements. These actually work as constants, as they do not change through the execution of the code. It is very likely that most of these positions will not be the same when you build your machine, so use the values here as guides only.

Line 14 sets the $zsafe to 0, which is at the tallest position (see main text for definitions of the zero positions). This ensures that any horizontal movement done at that position will be safe, that is, will not hit other objects.


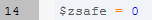


Lines 16 to 18 set the coordinates for the forceps placing the tin on the top of the balance plate.


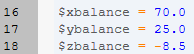


Lines 19 to 25 set the coordinates for the tin capsules on the tray. The start of a row or column is indicated by s, while the end is indicated by e. The number of cells is indicated by n. The vertical position of the tin capsules on the tray is $ztin.


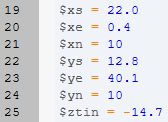


Lines 31 to 33 set the coordinates for the deposit tray. The pattern is similar to the sample tray shown previously.


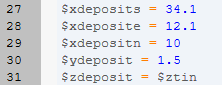


Lines 33 to 39 set the coordinates for the powder container when manipulated with the gripper. $xpowder1is the resting position for the container. $xpowder2s and e indicate the positions where the container delivers the powder to the tin. They are, actually, the cells indicated by $xdeposits and e. However, their values differ because the gripper will handle the container with the gripper, and not the forceps. $ypowder1 is the Y position at the rest position, and $ypowder2 is the Y position when delivering the powder. The first $zpowder, $zpowder1, is the coordinate for the container touching the tray. The other position, $zpowder2, is for the transfer of the container from $xpowder1 to $xpowder2. This coordinate is used instead of $zsafe to reduce powder being scattered during these transfers.


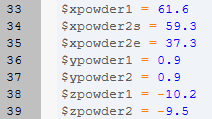


Lines 41 to 43 determine the positions for the gripper.


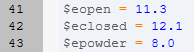


The first, $eopen, makes the forceps to be partially open, in a position that allows it to get to the tin capsules. The next, $eclosed, is the position that closes the forceps, and allows the grabbing of the tin capsule. The last, $epowder, is the position that ensures proper gripping of the powder container using the gripper (not the forceps).

Lines 45 to 54 create a matrix ($posxy) with the positions for the sample tray using the variables declared in lines 19 to 25.


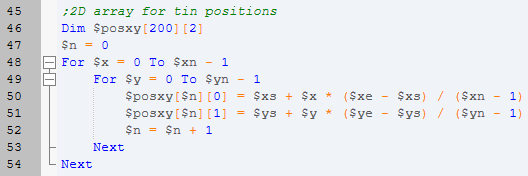


Lines 56 to 64 create two arrays ($xdeposit and $xpowder2) with the positions for the deposit tray using the variables declared in lines 27 to 39.


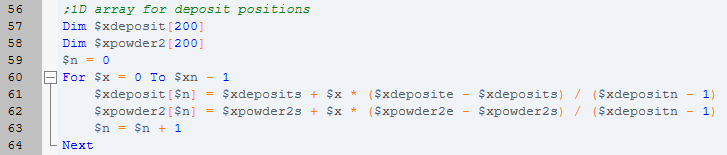


Lines 66 to 74 are the real acting part of the code, where the main function is called for each tin capsule. In the example shown, for the first 20 samples in the tray.


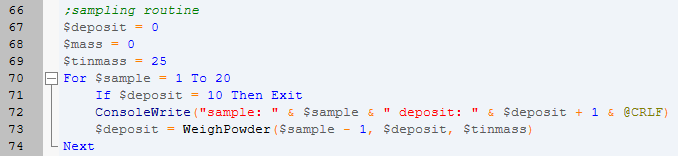


There are three variables declared here: $deposit, $tinmass and $sample. All three are needed for the function WeighPowder, which is the main function in the code. $tinmass gets an arbitrary value, 25, which is 25 mg in the real world (the tin capsules used here normally weigh more than 30 mg; 25 mg ensures that if a tin capsule is in the balance this will be noticed at all times). $deposit starts at 0, which is the standard starting array value for AutoIt. However, $sample starts at 1, which then needs to be corrected when input to WeighPowder (line 73). ConsoleWrite, at line 72, is called to register in the SciTE console what is happening in the code. Several other such instances will be present through the code.

The function WeighPowder is described in the following lines, 76-109. This is the most complex function in the code, and the actions in this function are better understood using section 6.2 as complementary explanation. In the next few paragraphs, an explanation of the function WeighPowder and only an overview of the several functions inside will be provided. In depth explanations about the functions are provided in latter paragraphs.


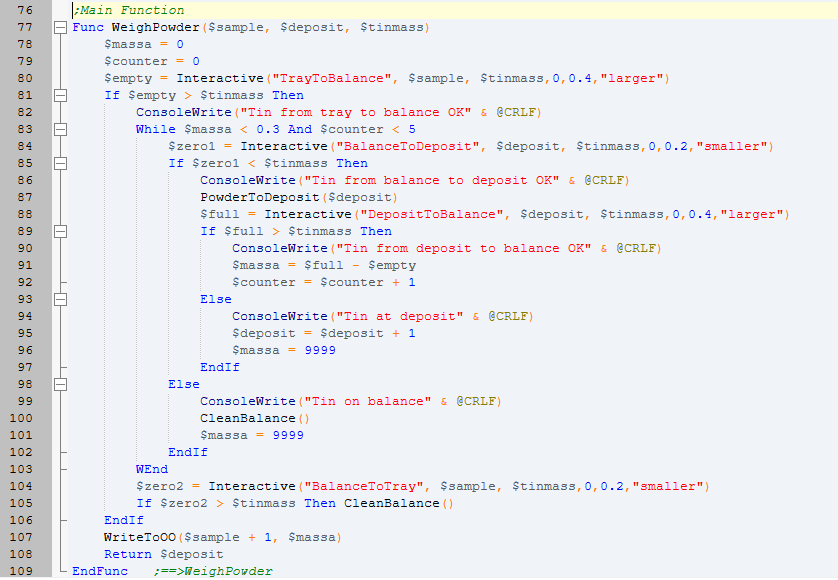


The first actions are the attribution of zero for the variables $massa and $counter. Then, the function Interactive is called, receiving 6 arguments: the function TrayToBalance, the variables $sample, $tinmass (both obtained from the input provided to WeighPowder), and the values 0, 04 and “larger”. This function returns the value $empty, which corresponds to the mass of the empty tin capsule. As explained before, this mass is typically above 30 mg. This mass is then compared to $tinmass (line 69). This is a test to determine if miau has correctly placed an empty tin capsule at the beginning of the weighing procedure. If not, the code jumps to line 106, where the function WriteToOO writes the sample and the variable $massa to an OpenOffice Calc spreadsheet. After that, the function WeighPowder returns the variable $deposit, which is used in line 71 to determine whether the code proceeds or not.

If $empty > $tinmass, then a message is displayed, indicating that a tin capsule was transferred from the tray to the balance. After that a While loop starts, testing the variable $massa against the value 0.3. The loop continues as long as $mass < 0.3, which is the minimum useful sample amount for our purposes. As the initial $massa value is zero, the loop proceeds. The next action, line 84, is to transfer the tin from the balance to the deposit (as in line 80, the function Interactive is called, but taking different arguments this time), returning the variable $zero1, which is the reading of the balance without any tin capsule on it. This value must be less than $tinmass. If not, the code jumps to line 99, where an indication is displayed indicating that the tin remained on the balance. The variable $massa gets a value of 9999, which is an impossible value but higher than 0.3. This will make the While loop stop. Then the code goes to line 104, where a last attempt to remove the tin capsule is performed (function Interactive with function BalanceToTray as argument). Failing that, a procedure to clean the balance is performed (line 105).

If $zero < $tinmass, a message is displayed indicating that the tin capsule was removed from the balance and is now at the deposit (line 86). The function PowderToDeposit is called, and then the function Interactive again, receiving DepositToBalance as argument, returning the variable $full. Again, a check is performed to verify if the tin capsule was correctly placed on the balance dish. If yes, another message is written registering the success (line 90). Then, the variable $massa receives the difference between variables $full and $empty, which is the mass of powder in the tin. Next, $counter is incremented by 1. If $massa is less than 0.3 and $counter reaches 5, the loop ends. If the tin is not placed on the balance, the code jumps to line 92, a notice of the error is displayed, the variable $deposit is incremented by 1, the variable $massa receives the value of 9999, resulting in a similar procedure as explained in the previous paragraph.

After the main function, the code lists all the subfunctions that build the main function. The first is Interactive (lines 112-130), which is an interactive procedure to repeat movements in case things go wrong. The main concept here is to retry the movement using the balance reading as a guide.


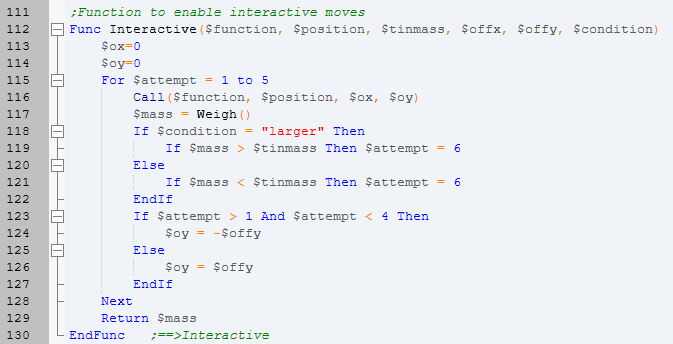


This function takes 6 arguments. The first is $function, which is one of the four functions that contains a series of movements for miau. These four functions are listed after this one. The next variable is $position, which can be $sample, if it refers to the sample tray, or $deposit, if it refers to the deposit tray. Then there is $tinmass, which in this code has a value of 25. The next two, $offx and $offy, are the variables that enable offsets for X and Y movements respectively. The last argument is $condition, which can be “larger” or “smaller”.

Inside the function, the first two lines attribute zero to two variables, $ox and $oy. Then a loop with 5 steps starts. The first action is to call the function passed as argument, with $position, $ox and $oy used as arguments for $function (the next 4 functions receive three arguments, see ahead in the text). Then, the variable $mass receives the value returned by the function Weigh, which is the reading of the balance (also presented ahead in the text). The next portion of the loop is an If statement dealing with $masa and $condition (lines 118 to 122). $condition can be “larger” or “smaller”. If it is “larger”, then there is another If test using the operator >. Else, the operator is <. The test compares $mass and $tinmass, and, depending on the outcome, makes attempt become 6, which ends the loop. This is a shortcut to end the loop in case there is no need for replications. The final portion of the loop is another If test (lines 123 to 127) in which the variable $oy is modified according to the loop step. Notice that the code does not modify $ox. This is because in the use of the autosampler so far, this has not been necessary. The option is kept open, though, and the code can be easily modified by adding another If statement inside the loop, increasing or reducing the number of steps in the loop, etc.

Lines 132 to 226 list functions that produce full moves, that is, movements that can be described as moving the tin or the powder container from point A to point B.

The first of such functions is TrayToBalance, which moves a tin capsule from its position in the tray to the balance plate. It takes as arguments $sample, which is the sample number, and two offset parameters, $offx and $offy. These offset parameters are used if a small modification in the movement is needed. Here, they are used when reaching the tin capsule in the tray (line 136).


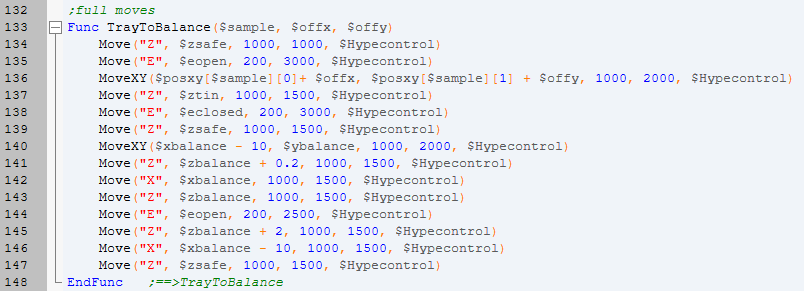


The function is a sequence of movements of the forceps (steps are line numbers): 134) move to safe vertical position; 135) open the forceps; 136) move horizontally to tin position in the tray; 137) move vertically down to tin position in the tray; 138) close the forceps; 139) bring to safe vertical position; 140) move horizontally to a position in front of the balance; 141) move to a vertical position close to that of the delivery on the balance plate; 142) move horizontally, only on the axis X inside the balance chamber; 143) move down a little to touch the tin to the balance place; 144) open the forceps to release the tin; 145) move up from the balance plate; 146) move horizontally out of the balance chamber; 147) move vertically to the safe position.

The next function is BalanceToTray, and it performs the opposite action of TrayToBalance. Notice that it also takes offsets as arguments, and these are called at lines 153 and 154.


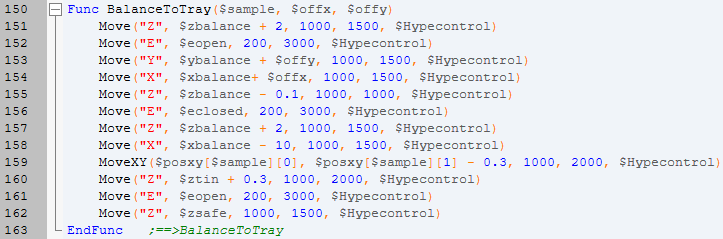


Commands: 151) bring the forceps to a position higher than the balance plate; 152) opens the forceps; 153) move to the Y position for the balance; 154) move inside the balance on the X axis; 155) move down to get the tin from the balance plate; 156) close forceps; 157) move up away from the balance plate; 158) move outside the balance chamber; 159) move to tin horizontal position in tray, a little more to the left; 160) move to a little above the tin vertical position in the tray; 161) open forceps to release tin; 162) move to safe vertical position.

The function DepositToBalance is very similar to TrayToBalance.


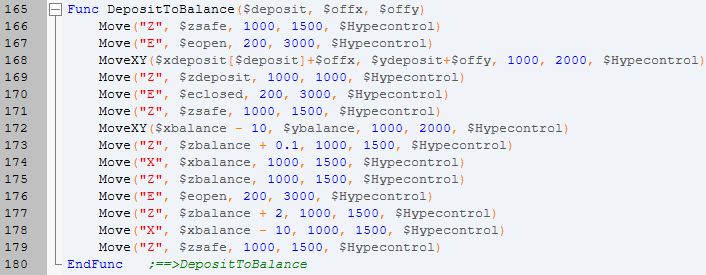


Accordingly, the function BalanceToDeposit is very similar to BalanceToTray.


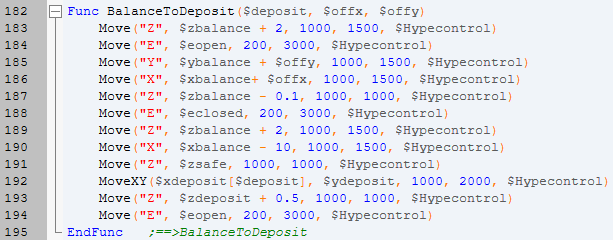


The next function, PowderToDeposit, differs from the previous ones in that it deals with the powder container, and not the forceps. Movements are similar to those in the previous functions.


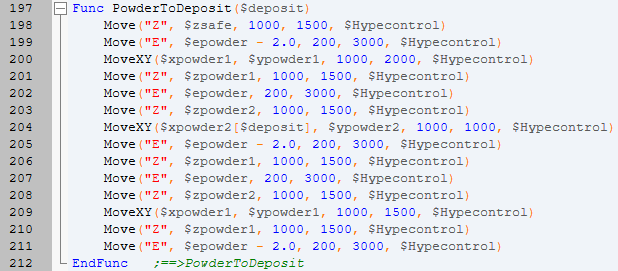


The last function in this category is CleanBalance. This function does not carry the tin capsule or the powder deposit. In fact, at line 216, the forceps are closed, and stay like that for the rest of the function. The movements here bring the closed forceps inside the balance chamber and make them move above the balance dish, so that if any tin capsule is there, it is pushed away.


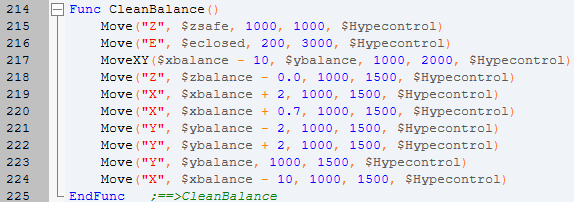


The functions above, except for the first two, were built by the functions in lines 227 to 240.


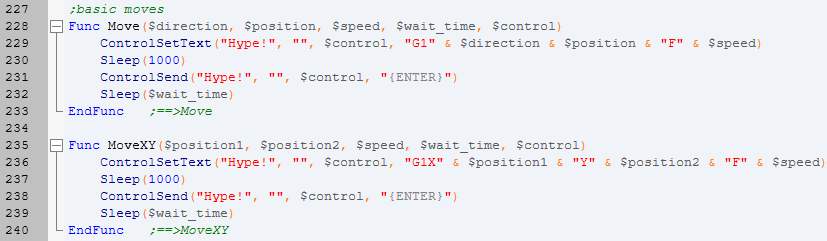


The first of them is Move, which takes 5 arguments: $direction, $position, $speed, $wait_time, and $control. $direction is the axis, which can be X, Y, Z or E. $position is the number of steps the motor will spin. The values are often those variables listed in lines 14 to 64. $speed is the feed rate. Here 1000 is used for axes X, Y and Z, and 200 for E. These values may change depending on the hardware that is used, so trial and error is advised for optimal results. $wait_time is the time in milliseconds allowed for the action to take place. $control is the control that receives the instructions in the function. It is always $Hypercontrol, the variable defined in line 5.

The function uses the command ControlSetText to send a text to Hype!Terminal which contains the instruction in G code (G1 command) for the control of the machine. Then a waiting time of 1 s is called (Sleep), and the ENTER key is sent using the command ControlSend. Another Sleep is called, this time using the $waiting_time passed as argument.

The next function, MoveXY, is very similar to Move. The difference is that now two position arguments are passed. This allow a faster horizontal movement, on a diagonal, instead of two successive Move commands that would go as two perpendicular movements.

In addition to the movement functions, there are functions dealing with weighing, listed in lines 242 to 267. Notice that these functions work for the specific balance used here. A different balance may need different functions to deal with their output.

The first function, GetNumberFromArray, does not interact with the balance directly, but is used to parse the value when reading its response. It sets the variable $mass to -2, an impossible value. Then, it subtracts 1 from each element of the array. If the obtained value of this operation is different from -1, then the value is assigned to the variable $mass.


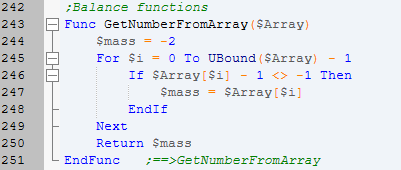


The next function is the Weigh function, which makes the balance return the mass present in it.


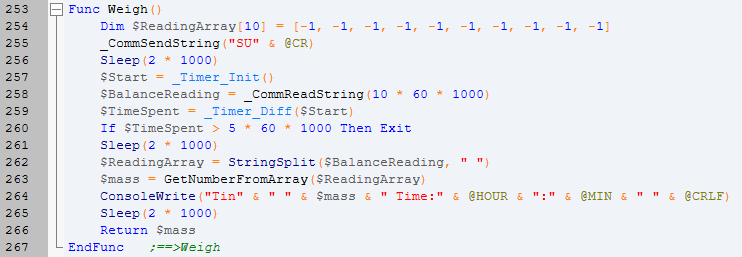


The first step is to create an array with 10 positions, all of them equal to -1. This is an artifice to parse the values obtained from the balance. Then, inside the loop, the first instruction is to send SU to the balance. This tells the balance to send the its stable reading in mg units. Wait 2 seconds, then a timer is started, followed by the _CommReadString function. The function _CommReadString is a dynamic function, which waits until a response comes from the balance to the computer (full details in the book Practical Laboratory Automation Made Easy with AutoIt, Carvalho, 2016). Here, the maximum waiting time is set to 10 minutes. However, a timer is also set just before this function. If this timer records a waiting time of longer than 5 minutes, the script is aborted. This is a safety procedure to avoid miau to damage the balance by trying to get the vial while the chamber is closed. In other words, if the balance takes longer than 5 minutes to stabilize and assume the zero value, the run is aborted. It is possible that the script waits up to ten minutes as the function _CommReadString works, but any value longer than 5 minutes results in cancelling the script. The value obtained with _CommReadString, $BalanceReading, is then converted to an array, $ReadingArray, using the command StringSplit. Then, this array is used as input to obtain the final mass value through the function GetNumberFromArray. Finally, the $mass value is returned.

The last function in the code is a function to store the weighed masses as values in the cells of an Open Office Calc spreadsheet.


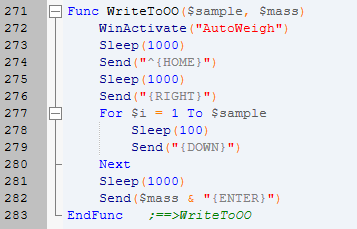


The spreadsheet needs to have a name starting with “AutoWeigh”, so that its window will be activated as in line 272. The next steps are simply keyboard inputs in order that the value is typed in the correct cell. Example of spreadsheet with useful name:


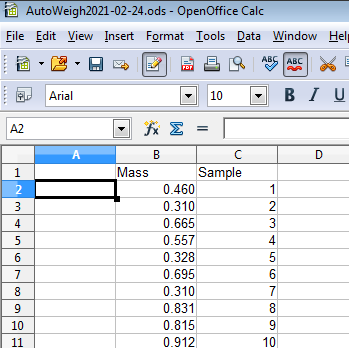

Supplement: Supplementary data 2 [file mmc2.docx]
